# Knockdown of CD146 promotes endothelial-to-mesenchymal transition via Wnt/β-catenin pathway

**DOI:** 10.1371/journal.pone.0273542

**Published:** 2022-08-24

**Authors:** Zhao-Yu Zhang, Chao Zhai, Xue-Yuan Yang, Hai-Bing Li, Li-Ling Wu, Li Li

**Affiliations:** 1 Department of Physiology and Pathophysiology, School of Basic Medical Sciences, Peking University Health Science Center, Beijing, China; 2 Key Laboratory of Molecular Cardiovascular Science, Ministry of Education, Beijing, China; 3 Beijing Key Laboratory of Cardiovascular Receptors Research, Beijing, China; "INSERM", FRANCE

## Abstract

**Purpose:**

Cardiac fibrosis is characterized by the excessive deposition of extracellular matrix (ECM) proteins and leads to the maladaptive changes in myocardium. Endothelial cells (ECs) undergoing mesenchymal transition contributes to the occurrence and development of cardiac fibrosis. CD146 is an adhesion molecule highly expressed in ECs. The present study was performed to explore the role of CD146 in modulating endothelial to mesenchymal transition (EndMT).

**Methods:**

C57BL/6 mice were subjected to subcutaneous implantation of osmotic minipump infused with angiotensin II (Ang Ⅱ). Adenovirus carrying CD146 short hairpin RNA (shRNA) or CD146 encoding sequence were infected into cultured human umbilical vein endothelial cells (HUVECs) followed by stimulation with Ang II or transforming growth factor-β1 (TGF-β1). Differentially expressed genes were revealed by RNA-sequencing (RNA-Seq) analysis. Gene expression was measured by quantitative real-time PCR, and protein expression and distribution were determined by Western blot and immunofluorescence staining, respectively.

**Results:**

CD146 was predominantly expressed by ECs in normal mouse hearts. CD146 was upregulated in ECs but not fibroblasts and myocytes in hearts of Ang II-infused mice and in HUVECs stimulated with Ang Ⅱ. RNA-Seq analysis revealed the differentially expressed genes related to EndMT and Wnt/β-catenin signaling pathway. CD146 knockdown and overexpression facilitated and attenuated, respectively, EndMT induced by Ang II or TGF-β1. CD146 knockdown upregulated Wnt pathway-related genes including *Wnt4*, *LEF1*, *HNF4A*, *FOXA1*, *SOX6*, and *CCND3*, and increased the protein level and nuclear translocation of β-catenin.

**Conclusions:**

Knockdown of CD146 exerts promotional effects on EndMT via activating Wnt/β-catenin pathway and the upregulation of CD146 might play a protective role against EndMT and cardiac fibrosis.

## 1. Introduction

Cardiac fibrosis, a requisite component that underlies nearly all forms of heart failure, is caused by a variety of damaging factors, e.g., pressure overload, ischemia, and metabolic disorders. It is characterized by excessive deposition of extracellular matrix (ECM) proteins and distorts the architecture of myocardium [1–3]. Endothelial to mesenchymal transition (EndMT) is a process whereby endothelial cells (ECs) convert into a mesenchymal phenotype induced by growth factors, hypoxia, metabolic disorders, inflammation, mechanical stress, etc. Accumulating evidence have demonstrated that cardiac fibroblasts/myofibroblasts derived from ECs contribute to the occurrence and development of cardiac fibrosis [4].

CD146, also known as melanoma cell adhesion molecule (MCAM) or Mucin18 (MUC18), is an adhesion molecule highly expressed in tumors and blood vessel-constituting cells predominantly ECs [5]. Human mature CD146 protein contains an extracellular region composed of 5 immunoglobulin-like domains, a hydrophobic transmembrane region and a short intracytoplasmic tail [6, 7]. It is actively involved in cell-cell and cell-ECM interactions and participates in biological processes including cell growth, proliferation, differentiation, migration and apoptosis [8]. Besides its adhesion properties, membrane-bound CD146 also acts as a cell-surface receptor to bind with miscellaneous ligands and mediate cellular signaling transduction [9]. Abnormal expression of CD146 has been implicated in the development and progression of cancers, autoimmune diseases, and cardiovascular diseases [10, 11].

Recent studies investigated the correlation between soluble CD146 (sCD146) and heart failure. sCD146, generated from the cleavage of membrane-bound CD146 by metalloproteases (MMPs) [12], has been identified as a novel circulating marker reflecting venous congestion [13, 14]. Plasma levels of sCD146 are increased in patients with acute coronary syndrome (ACS) showing signs of pulmonary edema and associated with the severity of pulmonary congestion [15]. Another study shows that sCD146 levels are higher in dyspnea patients with acutely decompensated heart failure (ADHF) than those non-cardiac dyspnea patients and correlate with echocardiographic markers of venous congestion [16]. In chronic heart failure patients, circulating sCD146 levels are rapidly and profoundly increased in the congested arm caused by a pressurized tourniquet cuff, compared to the uncompressed arm [17]. An ADHF rat model using thoracic aortic constriction indicates that CD146 is expressed in the intima of large arteries and associates with left ventricular function and organ congestion [18]. Above studies focus on extra-cardiac CD146 and evaluate its role as a biomarker in congestive heart failure, however, little is known about regional CD146 expression in heart and its effect on cardiac fibrosis.

In the present study, we investigated the expression profile of CD146 in hearts of angiotensin II (Ang II)-infused mice. We also determined the role of CD146 in modulating EndMT and explored the underlying mechanisms in cultured human umbilical vein endothelial cells (HUVECs).

## 2. Materials and methods

### 2.1 Materials

Mouse cardiac microvascular endothelial cells (MCMECs) (CP-M129) were purchased from Procell Life Science & Technology Co., Ltd. (Wuhan, China). Ang II (A9525) was purchased from Sigma-Aldrich Co. (St. Louis, MO, USA). Collagenase Ⅱ (LS004176) was from Worthington Biochemical Corporation (Lakewood, NJ, USA). Endothelial cell medium (ECM) (1001) was from ScienCell Research Laboratories (San Diego, California, USA). Antibodies for CD146 (ab87342), CD31 (ab76533), VE-Cadherin (ab33168), fibroblast specific protein-1 (FSP-1) (ab93283), β-catenin (ab68183), cardiac troponin Ⅰ (cTnⅠ) (ab92408) and CD68 (ab955) were from Abcam (Cambridge, MA, USA). Antibodies for α-smooth muscle actin (α-SMA) (14395-1-AP) and myeloperoxidase (MPO) (66177-1-Ig) were from ProteinTech Group (Chicago, IL, USA). Antibody for Snail (MAK089Hu21) was from Cloud-Clone Corp. (Beijing, China). Antibody for Slug (A1057) was from ABclonal Technology Co., Ltd (Wuhan, China). Antibody for glycogen synthase kinase-3β (GSK-3β) (bsm-33293M) was from Bioss (Beijing, China). Antibodies for platelet-derived growth factor receptor α (PDGFRα) (PA5-34739), CD4 (MA1-146) and CD19 (14-0194-82) were from Thermo Fisher Scientific (Waltham, MA, USA).

### 2.2 The mouse model of Ang II infusion

All experimental procedures were approved by the Ethics Committee of Animal Research, Peking University Health Science Center, and the investigation conformed to the Guide for the Care and Use of Laboratory Animals published by the US National Institutes of Health (NIH Publication No. 85–23, revised 1996). Male C57BL/6 mice aged 8–9 weeks were anesthetized with 2% isoflurane, placed on a heating pad, and received a single subcutaneous injection buprenorphine (0.1 mg/kg). Mice were then subcutaneously implanted with Alzet mini-osmotic pumps (Alza Pharmaceutics, Palo Alto, CA) containing either normal saline or Ang II (1.4 mg/kg per day for 14 or 28 days). Systolic blood pressure was measured under conscious state by the noninvasive tail-cuff method using the CODA blood pressure system (Kent Scientific, Torrington, CT) at 1 day before and 7, 14, 21, 28 days after surgery. At day 14 or 28, mice were euthanized with carbon dioxide and hearts were excised for further biochemical and histological analysis.

### 2.3 Isolation of cardiac myocytes (CMs) and fibroblasts (CFs) from the adult mouse heart

Protocol for isolating adult mouse CMs and CFs was performed as described previously [19]. Briefly, the mouse heart was retrogradely perfused using a Langendorff’s apparatus with solution A (Tyrode’s solution containing 10 mmol/L taurine) for 5 min and then circularly perfused with solution E (solution A containing 250 U/ml collagenase II and 1 mg/ml BSA) for 25 min. Left ventricle was removed, cut into small pieces, resuspended in fresh solution E, and agitated in a water bath at 37°C for 5 min. The supernatant was collected, filtered, and centrifuged at 500 × g for 45 s to obtain CMs. As for adult mouse CFs, ventricles from 8–10 week male C57BL/6 mice were minced and digested with 0.1% collagenase II at 37°C for 6–8 cycles. Cells were collected and a single preplating step was used to allow fibroblasts to attach to culture plates. Non-adherent cells were removed and CFs were collected and lysed for further investigation.

### 2.4 Culture of HUVECs and MCMECs

HUVECs and MCMECs were cultured in collagen-coated cell culture dishes and the medium was refreshed every 48 hours. All cells were maintained in 5% CO_2_ and 95% humidified air at 37°C. HUVECs at passages 4–6 and MCMECs at passages 1–2 were used for experiments.

### 2.5 Transfection of recombinant adenovirus carrying interested genes

Adenovirus carrying CD146 short hairpin RNA (Ad-shCD146) or CD146 encoding sequence (Ad-CD146) were designed and constructed by Likori Co., Ltd. (Beijing, China). Adenovirus carrying empty vector (Ad-EV) or scrambled shRNA (Ad-shScr) were used as negative controls for Ad-CD146 and Ad-shCD146, respectively. HUVECs were transfected with Ad-shCD146 (MOI = 100) or Ad-CD146 (MOI = 50) for 48 h to knockdown or overexpress CD146 expression, respectively.

### 2.6 Western blot analysis

Heart tissues or cells were homogenized with RIPA lysis buffer supplemented with protease inhibitors. Equal amounts of proteins were separated by SDS-PAGE and transferred to polyvinylidene difluoride (PVDF) membrane. Membranes were blocked with 5% non-fat milk and then incubated with primary antibodies overnight at 4°C. Then membranes were incubated with secondary antibodies conjugated with horseradish peroxidase for 1.5 h at room temperature. Blots were visualized with an enhanced chemiluminescence kit (Beijing Biodragon Immunotechnologies Co., Ltd). Images were captured by ChemiDocTM XRS+ (Bio-Rad) with Image Lab software. The densities of bands were quantified using Image J software.

### 2.7 Quantitative real-time polymerase chain reaction (qRT-PCR) analysis

Total RNA of heart tissues or cells was isolated by use of TRIzol Reagent (15596026, Thermo Fisher Scientific) and cDNA was synthesized from total RNA by use of the HiScript Ⅲ RT Supermix (R323-01, Vazyme Biotech Co., Ltd, Nanjing, China). qRT-PCR involved the use of SYBR green fluorescence (Q711-02, Vazyme Biotech Co., Ltd) and gene specific primers. The primers used in qRT-PCR were listed in S1 Table. All data were quantified by the use of the comparative cycle threshold method, normalized to GAPDH.

### 2.8 Morphological examination

De-paraffined sections (6 μm thickness) were stained with hematoxylin and eosin (H&E) staining, Masson’s trichrome staining, and Sirius Red staining, respectively. Microscopy images were captured using the Leica 550IW system (Leica, Mannheim, Germany). Myocyte cross-sectional area (CSA) and percentage of connective tissue were assessed by Image J software on H&E- and Masson’s trichrome-stained tissue specimens, respectively.

### 2.9 Immunofluorescence staining

De-paraffinized heart sections or HUVECs on coverslips were stained with primary antibodies overnight at 4°C, and then secondary antibodies conjugated with fluorescent-dye for 2 h at room temperature. Nuclei were counterstained with 4,6-diamidino-2-phenylindole (DAPI, MBD0015, Sigma-Aldrich). Fluorescence images were captured by Leica TCS SP8 confocal fluorescence microscopy.

### 2.10 RNA-sequencing analysis

HUVECs were transfected with Ad-shScr or Ad-shCD146 for 48 h and total RNA was extracted using TRIzol Reagent. The mRNA was enriched and processed into short fragments, which were used to synthesize cDNA. Sequencing adaptors were added to cDNA after purification and extraction. The quality control was performed by ND-2000 (NanoDrop Technologies). Sequencing was performed on Illumina Hiseq X Ten/ NovaSeq 6000 platform and assembled by Majorbio Bio-Pharm Technology Co., Ltd. (Shanghai, China). Differentially expressed genes were identified by filtering through log2 ratio (≥1) and FDR (≤0.05). Heatmaps were plotted at Majorbio.com online.

### 2.11 Statistical analysis

Data were reported as mean ± SEM. Two-tailed Student’s *t* test was performed to determine the difference between two groups. For multiple groups, differences were analyzed by one-way ANOVA followed by Bonferroni post hoc analysis. All the data were analyzed using GraphPad Prism 8.0 software. *P*<0.05 was considered statistically significant.

## 3. Results

### 3.1 CD146 is predominantly expressed in ECs of adult mouse hearts

**Fig 1:**
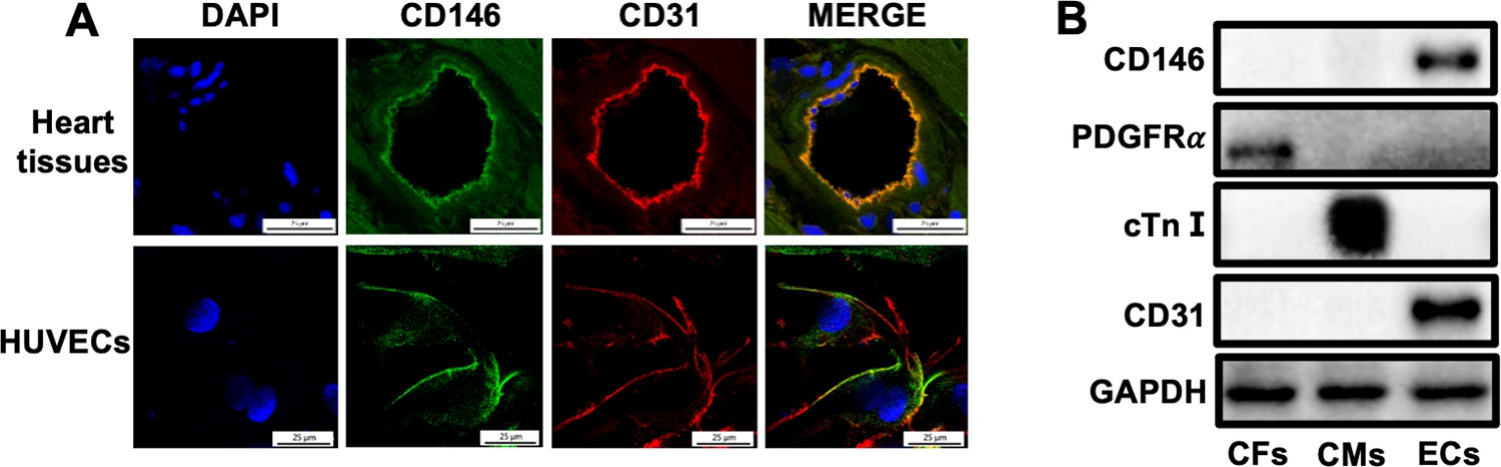
CD146 is predominantly expressed in ECs of adult mouse hearts. **A** CD146 expression in adult mouse hearts and HUVECs was determined by immunofluorescence staining (scale bars indicate 25 μm). Green represents CD146; red represents CD31; blue represents nuclei. **B** CD146 protein expression in CFs, CMs and ECs isolated from adult mouse hearts was detected by Western blot analysis. GAPDH served as an internal control. CFs: cardiac fibroblasts, CMs: cardiac myocytes, ECs: endothelial cells, cTnⅠ: cardiac troponin Ⅰ.

Heart sections or HUVECs were double stained with antibodies against CD146 and CD31 (a marker for ECs) and immunofluorescence staining results showed an abundant colocalization of CD146 with CD31 (Fig 1A). We then isolated CMs, CFs and ECs from adult mouse hearts. Western blot analysis revealed that CD146 was predominantly expressed in cardiac ECs but not in myocytes and fibroblasts (Fig 1B).

### 3.2 CD146 is upregulated in the fibrotic mouse hearts

**Fig 2:**
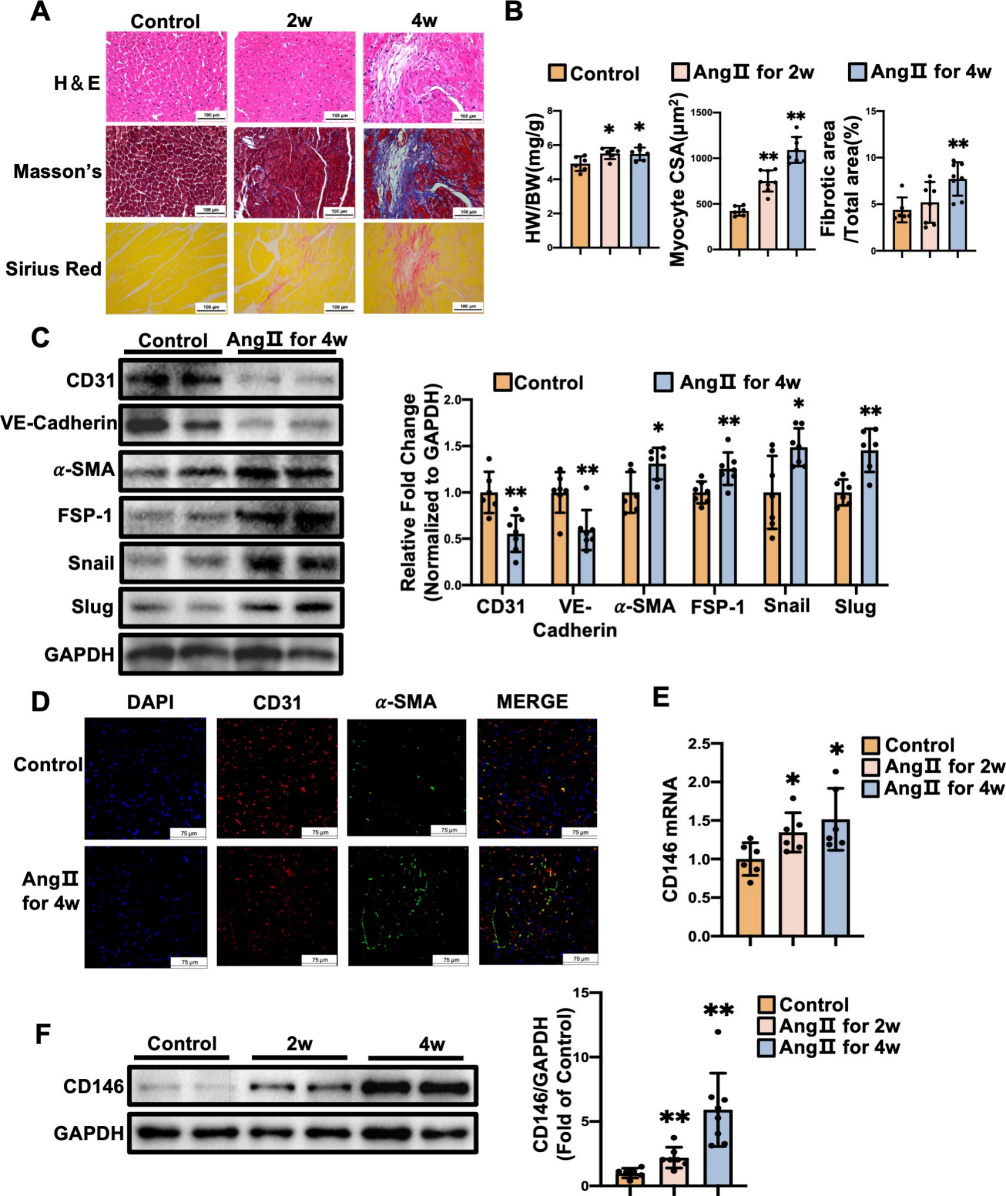
CD146 is upregulated in the fibrotic mouse hearts. **A** Mice were subjected to Ang Ⅱ (1.4 mg/kg/day) infusion for 2 and 4 weeks. Representative images of H&E staining, Masson’s trichrome staining, and Sirius Red staining (scale bars indicate 100 μm). **B** The ratio of heart weight to body weight (HW/BW) of mice was calculated. Myocyte cross–sectional area (CSA) and connective tissue percentage were measured from images captured from H&E–stained and Masson’s trichrome–stained sections, respectively. **C** The protein levels of CD31, VE–Cadherin, α–SMA, FSP–1, Snail, and Slug in left ventricles from control and Ang Ⅱ–infusion groups for 4 weeks were measured by Western blot analysis. GAPDH served as an internal control. **D** Representative double–immunofluorescence images of CD31 and α–SMA expression in left ventricles from control and Ang Ⅱ–infusion groups for 4 weeks (scale bars indicate 75 μm). Red represents CD31; green represents α–SMA; blue represents nuclei. Levels of CD146 mRNA (**E**) and protein (**F**) in left ventricles from control, Ang Ⅱ for 2 weeks, and Ang Ⅱ for 4 weeks. GAPDH served as an internal control. *P<0.05, **P<0.01 vs. Control.

Ang II infusion mice displayed elevated systolic and diastolic blood pressure (S1 Fig). Mice subjected to Ang II infusion for 2 and 4 weeks developed cardiac hypertrophy, as revealed by the rise in heart weight to body weight ratio (HW/BW) (*P*<0.01) and cardiac myocyte CSA (*P*<0.05). The fibrotic area was increased markedly in the mice infused with Ang Ⅱ for 4 weeks compared with the control mice (*P*<0.01) (Fig 2A and 2B), indicating the development of cardiac hypertrophy and fibrosis by Ang II treatment. We then examined the expression of EndMT markers and EndMT-inducing transcription factors at the 4-week time point. Ang Ⅱ-infusion mice exhibited the decreased levels of endothelial markers CD31 (*P*<0.01) and VE-Cadherin (*P*<0.01), the increased levels of mesenchymal markers α-SMA (*P*<0.05) and FSP-1 (*P*<0.01), and the elevated expression of EndMT-inducing transcription factors Snail (*P*<0.05) and Slug (*P*<0.01) in left ventricles, when compared to the saline-infusion mice (Fig 2C). Immunofluorescence staining showed an increased cell numbers co-expressing CD31 and α-SMA in heart tissues from Ang Ⅱ-infused mice (Fig 2D). These data indicate that Ang Ⅱ induces EndMT which contributes to cardiac fibrosis.

We then examined the expression profile of CD146 in left ventricles of mice infused with Ang II. The mRNA and protein levels of CD146 in left ventricles were increased at 2 and 4 weeks after Ang Ⅱ infusion (Fig 2E and 2F). These data suggest that CD146 is upregulated during the process of EndMT and cardiac remodeling.

### 3.3 CD146 upregulation by Ang II is mainly restricted to cardiac ECs

**Fig 3:**
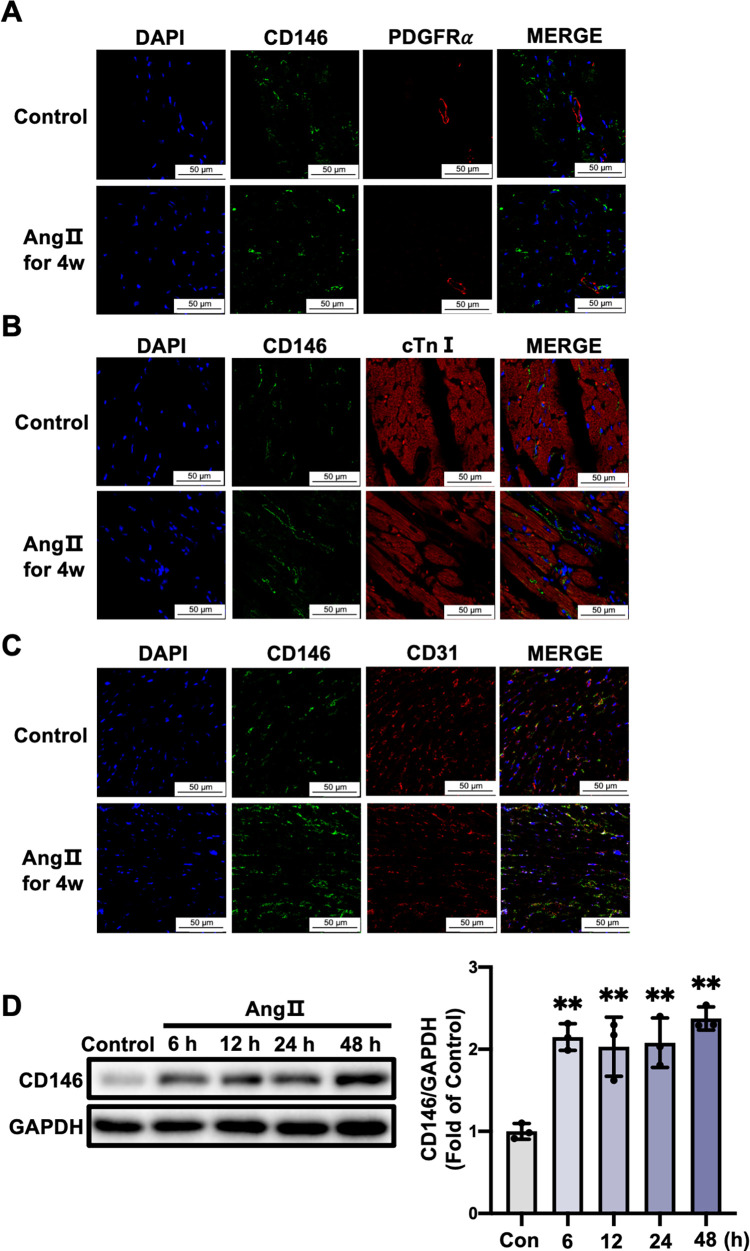
CD146 upregulation by Ang II is mainly restricted to cardiac ECs. Representative double–immunofluorescence images of CD146 with PDGFRα (**A**), cTnⅠ (**B**) and CD31 (**C**) in left ventricles from control and 4–week Ang II–infused mice (scale bars indicate 50 μm). Green represents CD146; red represents PDGFRα, cTnⅠ and CD31, respectively; blue represents nuclei. **D** HUVECs were treated with Ang Ⅱ (1×10^−6^ mol/L) for the indicated times and the protein levels of CD146 were measured by Western blot analysis. GAPDH served as an internal control. *P<0.05, **P<0.01 *vs*. Control.

To reveal the cell types responsible for the increased CD146 expression in Ang II-infused mice, heart sections were double stained with antibodies against CD146 and cardiac troponin I (cTnI, a marker for cardiac myocytes), platelet derived growth factor receptor α (PDGFRα, a marker for fibroblasts) and CD31, respectively. Results showed apparent colocalization of CD146 with CD31 in heart tissues from control and Ang II-infused mice. No colocalization with cTnI or PDGFRα was observed (Fig 3A–3C). We also detected whether CD146 was expressed in smooth muscle cells (SMCs) and certain immune cells in heart tissues. Immunofluorescence staining results showed that CD146 was not colocalized with α-SMA (a marker for SMCs), MPO (a marker for neutrophils), CD68 (a marker for monocytes/macrophages) and CD19 (a marker for B *lymphocytes*), and sparsely colocalized with CD4 (a marker for T-helper cells) (S2 Fig). These results indicate that ECs is the major cell type for cardiac CD146 upregulation in Ang II-infused mice. We then examined the effect of Ang II on CD146 expression in HUVECs. Western blot analysis showed that the expression of CD146 in HUVECs was upregulated after Ang Ⅱ stimulation for 6, 12, 24 and 48 h (*P*<0.01) (Fig 3D).

### 3.4 ECs with CD146 knockdown acquire a mesenchymal phenotype

**Fig 4:**
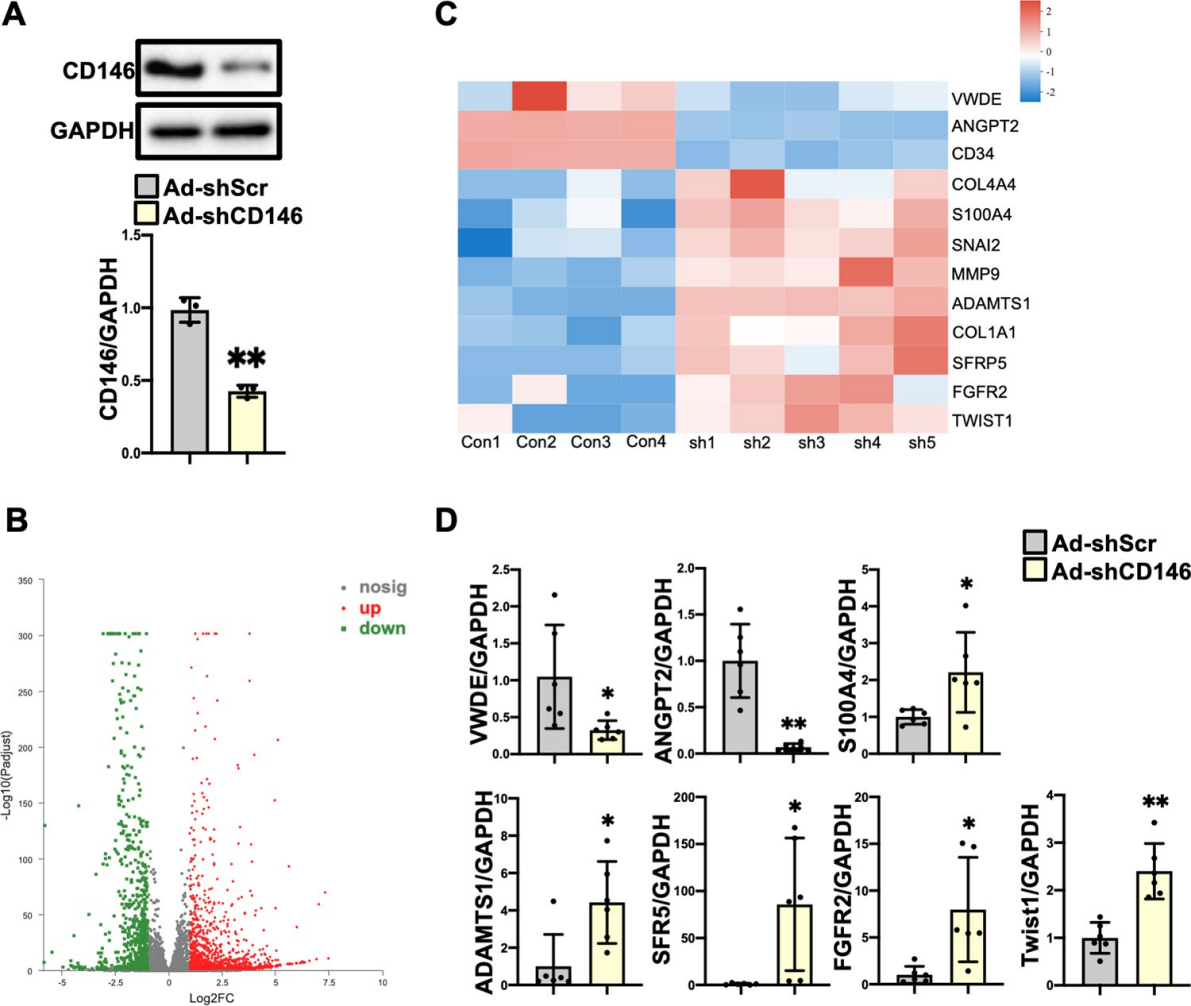
ECs with CD146 knockdown acquire a mesenchymal phenotype. **A** HUVECs were infected with Ad–shScr and Ad–shCD146 for 48 h. The protein level of CD146 was measured by Western blot analysis. GAPDH served as an internal control. **B** Numbers of differentially expressed genes in comparison of HUVECs infected with Ad–shScr and Ad–shCD146. **C** Heatmap depicting the differentially expressed genes related to EndMT. **D** The mRNA levels of *VWDE*, *ANGPT2*, *S100A4*, *ADAMTS1*, *SFR5*, *FGFR2*, and *Twist1* were measured by qRT–PCR analysis. *P<0.05, **P<0.01 *vs*. Ad–shScr. Ad–shScr: Ad–shScramble.

Next, we used RNA sequencing analysis to identify differentially expressed genes in ECs with CD146 knockdown. HUVECs were infected with Ad-shCD146 and the protein levels of CD146 were significantly decreased (*P*<0.01) (Fig 4A). Compared to ECs infected with Ad-shScr, 1890 differentially expressed genes were identified in ECs infected with Ad-shCD146, of which 1163 genes were upregulated and 727 genes were downregulated (Fig 4B, Excel_1 in S1 Data). Some differentially expressed genes resembled the signature of EndMT. Endothelial markers including *ANGPT2*, *CD34* and *VWDE* were downregulated and mesenchymal markers including *COL4A4*, *COL1A1*, *S100A4*, *ADAMTS1*, *SFRP5*, *FGFR2* and *MMP9* were upregulated. EndMT-inducing transcription factors including *SNAI2* and *Twist1* were also upregulated (Fig 4C, Excel_2 in S1 Data). We then detected the mRNA levels of above genes using qRT-PCR to confirm the transcriptome analysis differences. As shown in Fig 4D, the gene expression profiles of *VWDE* (*P*<0.05), *ANGPT2* (*P*<0.01), *SFR5* (*P*<0.05), *ADAMTS1* (*P*<0.05), *FGFR2* (*P*<0.05), *S100A4* (*P*<0.05) and *Twist1* (*P*<0.01) were consistent with the RNA-Seq results.

### 3.5 Knockdown of CD146 promotes Ang Ⅱ- and TGF-β1-induced EndMT

**Fig 5:**
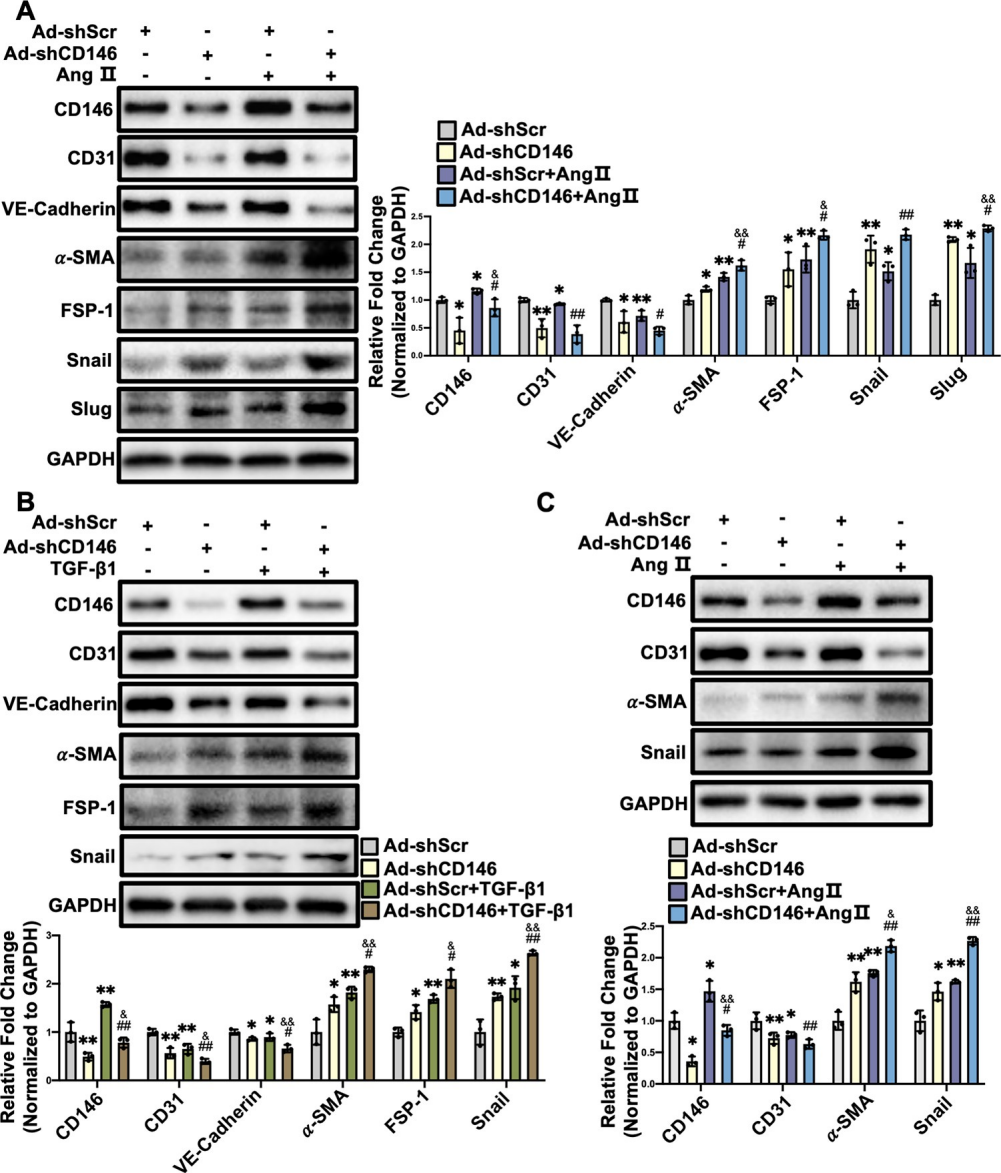
Knockdown of CD146 promotes Ang Ⅱ–and TGF–β1–induced EndMT. HUVECs were infected with Ad–shScr or Ad–shCD146 for 48 h followed by Ang Ⅱ (1×10^−6^ mol/L) (**A**) or TGF–β1 (10 ng/ml) (**B**) treatment for 48 h. The protein levels of CD146, CD31, VE–Cadherin, α–SMA, FSP–1, Snail and Slug were measured by Western blot analysis. GAPDH served as an internal control. **C** MCMECs were infected with Ad–shScr or Ad–shCD146 for 48 h followed by Ang Ⅱ (1×10^−6^ mol/L) treatment for 48 h. The protein levels of CD146, CD31, α–SMA and Snail were measured by Western blot analysis. GAPDH served as an internal control. *P<0.05, **P<0.01 *vs*. Ad–shScr, ^#^*P*<0.05, ^##^ *P*<0.01 *vs*. Ad–shScr+Ang Ⅱ or TGF–β1. ^&^*P*<0.05, ^&&^ *P*<0.01 vs. Ad–shCD146. Ad–shScr: Ad–shScramble.

To further confirm the potential role of CD146 in EndMT, HUVECs were infected with Ad-shScr or Ad-shCD146 and then incubated with Ang Ⅱ or TGF-β1, two potent factors driving EndMT and cardiac fibrosis. Ang II reduced the protein levels of endothelial markers (CD31 and VE-Cadherin) and induced the expression of mesenchymal markers (α-SMA and FSP-1) and EndMT-inducing transcription factors (Snail and Slug), which was further facilitated by CD146 knockdown (Fig 5A). CD146 knockdown also aggravated TGF-β1-induced EndMT (Fig 5B). HUVECs infected with Ad-shCD146 also exhibited lower levels of endothelial markers and higher levels of mesenchymal markers in the absence of Ang Ⅱ or TGF-β1 (Fig 5A and 5B). To further confirm whether CD146 knockdown exerts a similar effect in MCMECs, MCMECs were infected with Ad-shScr or Ad-shCD146, followed by Ang Ⅱ treatment for 48 h. Western blot analysis showed that knockdown of CD146 reduced CD31 expression and increased the protein levels of α-SMA and Snail under basal and Ang II-treated conditions (Fig 5C). Taken together, these results indicate that knockdown of CD146 promotes Ang Ⅱ- and TGF-β1-induced EndMT.

### 3.6 Overexpression of CD146 suppresses Ang Ⅱ- and TGF-β1-induced EndMT in HUVECs

**Fig 6:**
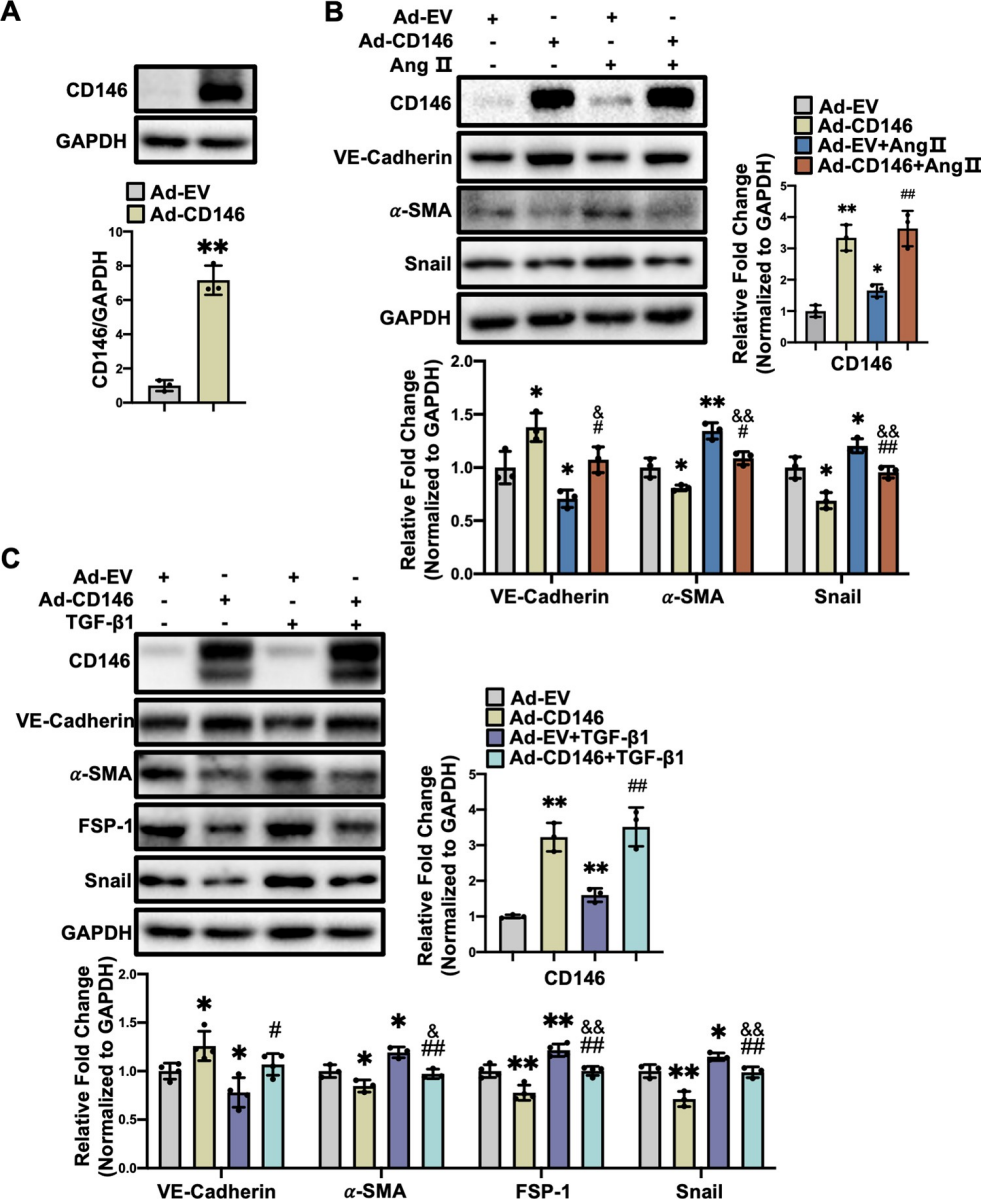
Overexpression of CD146 suppresses Ang Ⅱ–and TGF–β1–induced EndMT in HUVECs. **A** HUVECs were infected with Ad–EV and Ad–CD146 for 48 h. The protein level of CD146 was measured by Western blot analysis. GAPDH served as an internal control. HUVECs were infected with Ad–EV or Ad–CD146 for 48h followed by Ang Ⅱ (1×10^−6^ mol/L) (**B**) or TGF–β1 (10 ng/ml) (**C**) treatment for 48h. The protein levels of CD146, VE–Cadherin, α–SMA, FSP–1, and Snail were measured by Western blot analysis. GAPDH served as an internal control. *P<0.05, **P<0.01 *vs*. Ad–EV, ^#^*P*<0.05, ^##^ *P*<0.01 *vs*. Ad–EV+ Ang Ⅱ or TGF–β1. ^&^*P*<0.05, ^&&^*P*<0.01 *vs*. Ad–EV. Ad–EV: Ad–empty vector.

We then explored whether CD146 overexpression exerts protective effects against EndMT. HUVECs were infected with Ad-CD146 and the protein levels of CD146 were elevated markedly (*P*<0.01) (Fig 6A). Western blot analysis showed that overexpression of CD146 reversed the upregulation of mesenchymal markers and EndMT-inducing transcription factor Snail and the downregulation of VE-Cadherin induced by Ang Ⅱ (Fig 6B) and TGF-β1 (Fig 6C), indicating that CD146 overexpression attenuated EndMT induced by Ang Ⅱ and TGF-β1.

### 3.7 Knockdown of CD146 leads to the activation of canonical Wnt/β-catenin pathway in HUVECs

**Fig 7:**
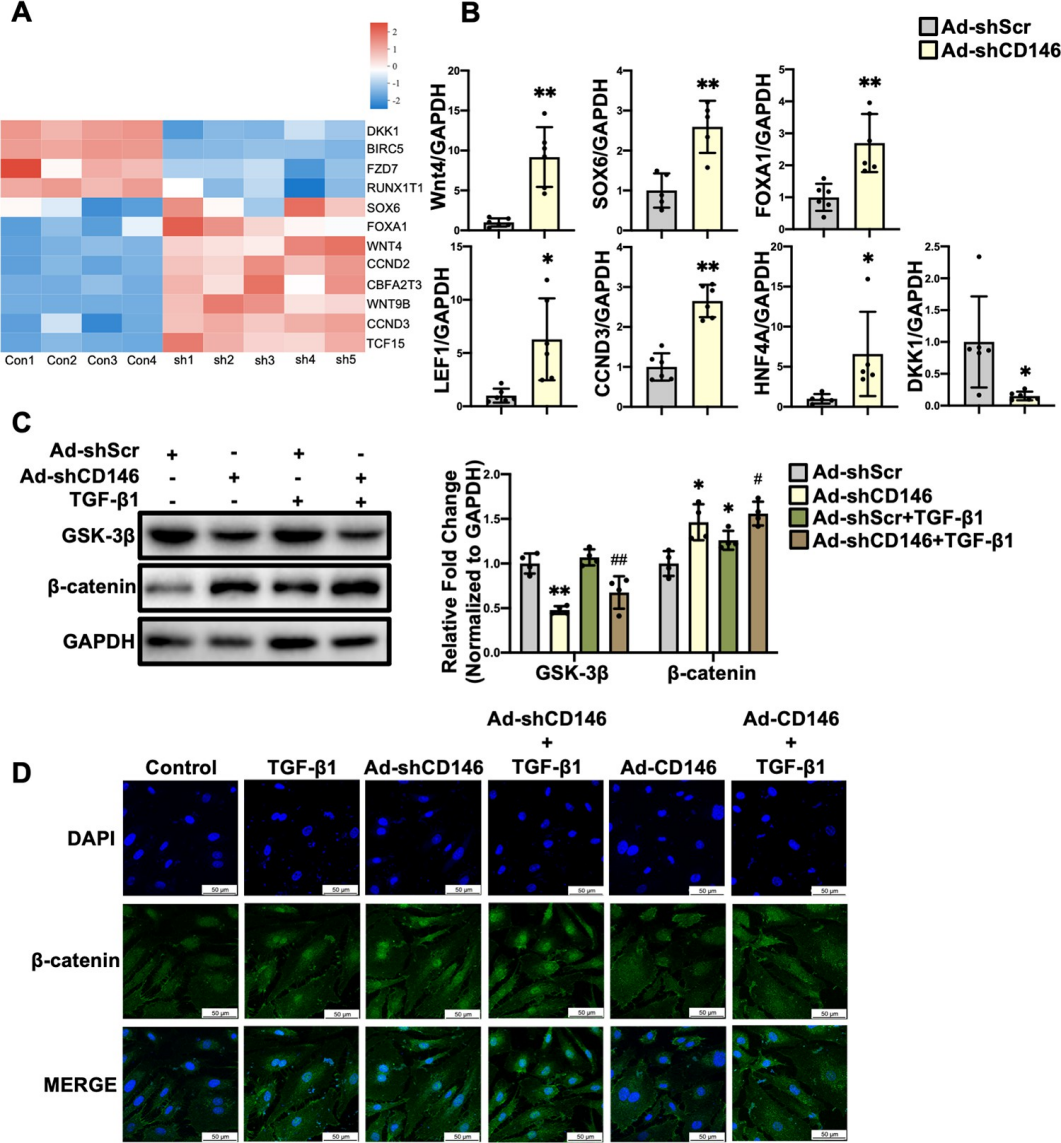
Knockdown of CD146 leads to the activation of canonical Wnt/β–catenin pathway in HUVECs. **A** Heatmap depicting the differentially expressed genes related to Wnt signaling pathway. **B** The mRNA levels of *Wnt4*, *SOX6*, *FOXA1*, *LEF1*, *CCND3*, *HNF4A* and *DKK1* were measured by qRT–PCR analysis. **C** HUVECs were infected with Ad–shScr or Ad–shCD146 for 48h followed by TGF–β1 (10 ng/ml) treatment for 48h. The protein levels of GSK–3β and β–catenin were measured by Western blot analysis. GAPDH served as an internal control. **D** HUVECs were infected with Ad–shCD146 or Ad–CD146 for 48 h followed by TGF–β1 (10 ng/ml) treatment for 6 h. Representative immunofluorescence images of β–catenin translocation in HUVECs (scale bars indicate 50 μm). Green represents β–catenin; blue represents nuclei. *P<0.05, **P<0.01 *vs*. Ad–shScr, ^#^*P*<0.05, ^##^*P*<0.01 *vs*. Ad–shScr+TGF–β1. Ad–shScr: Ad–shScramble.

We then explored the underlying mechanism by which CD146 knockdown facilitates TGF-β1-induced EndMT. Wnt/β-catenin signaling is considered a canonical pathway modulating EndMT [20]. Our RNA-Seq analysis showed that the Wnt signaling ligands including *Wnt4* and *Wnt9B*, Wnt pathway-related transcription factors including *SOX6*, *FOXA1* and *CBFA2T3*, and Wnt target genes including *CCND2*, *CCND3* and *TCF15* were upregulated while *DKK1* (the inhibitor of Wnt pathway) was downregulated in HUVECs with CD146 knockdown (Fig 7A, Excel_2 in S1 Data). qRT-PCR results also showed that *Wnt4* (*P*<0.01), *LEF1* (*P*<0.05), *HNF4A* (*P*<0.05), *FOXA1* (*P*<0.01), *SOX6* (*P*<0.01) and *CCND3* (*P*<0.01) were elevated while *DKK1* (*P*<0.05) was decreased in CD146 knockdown HUVECs (Fig 7B), which were in accordance with RNA-Seq analysis, suggesting that knockdown of CD146 leads to the activation of Wnt signaling pathway. *Wnt4* and *Wnt9B* have been implicated as activators of canonical Wnt signaling pathway [21], we therefore detected the protein level of β-catenin, a core component of the canonical Wnt signaling pathway. Western blot showed that β-catenin protein levels were increased in CD146 knockdown HUVECs (*P*<0.05) (Fig 7C). GSK-3β can lead to β-catenin phosphorylation and the subsequent ubiquitination and degradation by proteasome [22]. We found that the protein levels of GSK-3β were downregulated in CD146 knockdown HUVECs (*P*<0.01) (Fig 7C), suggesting that CD146 knockdown increased β-catenin protein level through manipulating GSK-3β. Upon Wnt stimulation, β-catenin was translocated into nucleus where it binds to the promoter of TCF/LEF to regulate the expression of target genes [23]. Therefore, the subcellular localization of β-catenin in HUVECs was examined. As shown in Fig 7D, TGF-β1 induced the nuclear translocation of β-catenin, which was further facilitated by CD146 knockdown while impeded by CD146 overexpression. Taken together, these results demonstrated that knockdown and overexpression of CD146 promoted and inhibited, respectively, EndMT via manipulating Wnt/β-catenin signaling pathway.

## 4. Discussion

Our results demonstrated that CD146 was predominantly expressed in ECs in adult mouse hearts. CD146 expression was upregulated in the heart of Ang Ⅱ-infused mouse model and in HUVECs stimulated by Ang Ⅱ. Knockdown of CD146 endowed ECs with mesenchymal phenotype while overexpression of CD146 restored endothelial phenotype in HUVECs. Furthermore, knockdown of CD146 upregulated Wnt ligands and increased cytosolic accumulation as well as nuclear translocation of β-catenin in HUVECs, suggesting that knockdown of CD146 activated canonical Wnt/β-catenin signaling pathway to promote EndMT.

Although originally identified as a melanoma cell adhesion molecule [7], CD146 has been detected in various cell types, including vessel constituting cells (ECs [5], pericytes [24], and SMCs [25]), cancer-associated fibroblasts [26], extravillous trophoblasts [27], mesenchymal stem cells [28] and lymphocytes [29]. In adult mouse heart tissues, immunofluorescence staining results indicated the predominant expression of CD146 in ECs, but not in CMs, CFs, SMCs and certain immune cells. We isolated ECs, CMs and CFs from the adult mouse heart and confirmed the abundant expression of CD146 in cardiac ECs. It has been documented that CD146 expression is regulated by a variety of factors. Examples are increased CD146 expression by IL-13 in airway epithelial cells [30], by endothelin-1 in melanocytes [31], by high glucose in renal tubular epithelial cells [32], and by oxidized-low density lipoprotein (ox-LDL) in macrophages [33]; and decreased CD146 expression by cigarette smoke extract (CSE) and IL-18 in pulmonary ECs [34], by miR-329 in HUVECs and human microvascular endothelial cells (HMECs) [35], and by miR-573 in melanoma cells [36]. Our study for the first time demonstrated that CD146 expression in ECs was elevated in left ventricles of Ang Ⅱ-infused mice and in HUVECs stimulated by Ang Ⅱ, suggesting that this pro-fibrotic factor can upregulate CD146 in ECs. Our findings are in accordance with previous studies showing upregulated CD146 by TGF-β in annulus fibrosus cells [37], cancer-associated fibroblasts [38], mouse primary hepatocytes and hepa 1–6 cells [39]. It is noteworthy that Ang II infusion for 4 weeks can induce ROS generation, macrophage infiltration, cell proliferation, and glomerular matrix deposition in the kidney [40], which contributes to arterial pressure regulation and cardiac remodeling [41, 42]. A prolonged exposure to inflammation and oxidative stress present in renal damage perturbs EC homeostasis and results in cardiac endothelial dysfunction [43, 44]. We therefore speculate that the changes in humoral homeostasis associated with kidney damage might influence EC function and gene expression profiles in the chronic Ang II infusion model.

Bardin et al. have shown that CD146 is upregulated during endothelial monolayer formation and correlates with a more cohesive structure and a decreased paracellular permeability [5]. The decrease of CD146 in rat pulmonary artery ECs was accompanied by increased permeability and enhanced macrophage infiltration. CD146 knockout mice exhibited aggravated inflammation and bronchoalveolar lavage fluid formation [34]. Conversely, another study demonstrated that CD146-deficient mice displayed a normal basal vessel permeability but a significant reduction in VEGF-induced vessel permeability [45]. In a mouse model of experimental cerebral malaria, upregulated endothelial CD146 facilitates the sequestration of red blood cells and lymphocytes and disrupts the integrity of blood-brain barrier [46]. These results indicate the distinct functions of CD146 on endothelial integrity and permeability under different pathological settings. Our RNA sequencing analysis as well as data from qPCR and Western blot analysis found that CD146 knockdown in HUVECs led to EndMT, characterized by the reduced expression of EC markers, the acquirement of mesenchymal markers, and the upregulation of EndMT-inducing transcription factors. The neurohumoral mediator Ang II is released in most cardiac pathological conditions and induces the transcription and secretion of TGF-β, thereby acts synergistically to promote the fibrotic response [47–49]. We therefore determined the role of CD146 in Ang II- and TGF-β1-induced EndMT. CD146 deficiency further facilitated whereas CD146 overexpression mitigated Ang II- and TGF-β1-induced EndMT, suggesting the inhibitory effect of CD146 on EndMT. A recent study showed that endothelial-specific deletion of CD146 alleviated EndMT and interstitial fibrosis in nephrotoxic serum-induced glomerulonephritis [50]. CD146 has also been identified as an inducer of epithelial to mesenchymal transition (EMT) and promotes cancer progression and metastasis [10]. The discrepancy between our results and the earlier findings indicates the complex and diverse functions of CD146 in different pathological conditions.

Our RNA-Seq data identified the upregulation of several Wnt ligands (*Wnt4* and *Wnt9B*) and Wnt signaling target genes (*CCND2*, *CCND3* and *TCF15*) in CD146-knockdown HUVECs. The increased expression of *Wnt4*, *LEF1*, *FOXA1*, *SOX6* and *CCND3* were further verified by qPCR. It has been documented that *Wnt4* and *Wnt9B* are ligands which mainly activate the canonical Wnt/β-catenin signaling pathway [21]. In the absence of Wnt, GSK-3β, Axin, and adenomatosis polyposis coli (APC) form a complex to induce the phosphorylation and cytoplasmic sequestration of β-catenin, thereby facilitate the ubiquitylation and proteasomal degradation of β-catenin. Upon Wnt stimulation, Wnt binds to the extracellular domains of both lipoprotein receptor related proteins (LRP) and Frizzled (FZD) receptors, which in turn induces the destabilization of Axin/APC/GSK-3β complex and stabilization of β-catenin. Nuclear translocation of β-catenin can drive the transcription of genes including *Snail*, *Slug*, and *Twist1* and lead to the occurrence of EndMT [23]. Growing evidence demonstrated the inhibitory effects of CD146 on canonical Wnt signaling components. CD146 inhibited FZ8-, LRP6- and dishevelled 2 (Dvl2)-activated canonical Wnt signaling through promoting β-catenin degradation in cytosol [51]. Another study showed that CD146 facilitated β-catenin degradation through promoting nuclear factor-κB (NF-κB)-initiated GSK-3β expression. Consistently, knockdown of CD146 in colorectal cancer (CRC) cells facilitated the transcriptional activation of β-catenin/TCF/LEF complex and thus endowed CRC cells with stem cell phenotype [52]. In the present study, the GSK-3β protein levels were reduced and the protein levels and nuclear localization of β-catenin were enhanced in CD146-knockdown HUVECs, suggesting the activation of Wnt/β-catenin signaling pathway by CD146 knockdown. Based on our findings that CD146 was upregulated in the fibrotic hearts and in Ang Ⅱ-treated HUVECs and CD146 overexpression inhibited β-catenin activation and EndMT, we speculate that upregulation of CD146 in ECs might be a compensatory mechanism protecting against EndMT and cardiac fibrosis.

There are several limitations that must be acknowledged in the current study. Firstly, although Ad-shCD146 infection results in a significant reduction of CD146 expression, CRISPR/Cas9-mediated stable knockout of CD146 in HUVECs could provide more convincing evidence of its pivotal role in EndMT. Secondly, there is no proof so far to support our assumption that upregulated CD146 in ECs alleviates cardiac fibrosis induced by Ang Ⅱ infusion *in vivo*. Transgenic mice with endothelial specific knockdown or overexpression of CD146 will be needed for further investigation. Thirdly, although the modulation of Wnt/β-catenin pathway by CD146 was observed in the current study, the key mechanisms by which CD146 regulates Wnt/β-catenin pathway need to be further explored.

## 5. Conclusions

We provided the evidence that CD146 was upregulated in the hearts of Ang Ⅱ-infused mouse and in HUVECs stimulated by Ang Ⅱ. Knockdown of CD146 promoted EndMT via activating canonical Wnt/β-catenin pathway. Our results indicated that CD146 plays an inhibitory role in EndMT and targeting CD146 might be a novel therapeutic strategy for cardiac fibrosis.

## Supporting information

S1 Table The primers used in qRT-PCR. The primers used in qRT-PCR are listed in S1 Table.
(DOCX)

S1 Fig Ang II infusion mice displayed elevated systolic and diastolic blood pressure. Mice were subjected to Ang Ⅱ (1.4 mg/kg/day) infusion for 2 (**A**) and 4 (**B**) weeks. Blood pressure was measured under conscious state by the noninvasive tail-cuff method using the CODA blood pressure system on the day of surgery and at 7, 14, 21, 28 days after surgery. *P<0.05 *vs*. 0 day, **P<0.01 *vs*. 0 day.
(DOCX)

S2 Fig CD146 was not expressed in SMCs and certain immune cells in adult mouse hearts. Representative double-immunofluorescence images of CD146 with α-SMA (a marker for SMCs), MPO (a marker for neutrophils), CD68 (a marker for monocytes/macrophages), CD19 (a marker for B lymphocytes), and CD4 (a marker for T-helper cells) in left ventricles from adult mice (scale bars indicate 50μm). Green represents CD146; red represents CD4, CD19, CD68, MPO and α-SMA, respectively; blue represents nuclei.
(TIF)

S1 Data RNA-Seq analysis were used to identify differentially expressed genes (DEGs) in ECs with CD146 knockdown. Total DEGs and DEGs related to EndMT as well as Wnt signaling pathway in ECs with CD146 knockdown were listed in Excel_1 and Excel_2, respectively.
(XLSX)

S1 Raw images The original uncropped and unadjusted images underlying all blot results. (PDF)
