# Supplementary material for: Knockdown of CD146 promotes endothelial-to-mesenchymal transition via Wnt/β-catenin pathway
Source: PLoS One. 2022 Aug 24;17(8):e0273542. doi: 10.1371/journal.pone.0273542 (PMC9401105; doi:10.1371/journal.pone.0273542)
Supplement: S1 Table — The primers used in qRT-PCR are listed in S1 Table. (DOCX) [file pone.0273542.s001.docx]

**Table S1. The primers used in qRT-PCR**

|  | Forward (5’-3’) | Reverse (5’-3’) |
| --- | --- | --- |
| GAPDH | GGAGCGAGATCCCTCCAAAAT | GGCTGTTGTCATACTTCTCATGG |
| CD146 | AGCTCCGCGTCTACAAAGC | CTACACAGGTAGCGACCTCC |
| CD31 | AACAGTGTTGACATGAAGAGCC | TGTAAAACAGCACGTCATCCTT |
| VE-Cadherin | GTTCACGCATCGGTTGTTCAA | CGCTTCCACCACGATCTCATA |
| 𝛼-SMA | GGCATTCACGAGACCACCTAC | CGACATGACGTTGTTGGCATAC |
| FSP-1 | TGAGCAACTTGGACAGCAACA | CTTCTTCCGGGGCTCCTTATC |
| Snail | GAAAGGCCTTCTCTAGGCCC | TCTTGGTGCTTGTGGAGCAA |
| Slug | CGAACTGGACACACATACAGTG | CTGAGGATCTCTGGTTGTGGT |
| ANGPT2 | AACTTTCGGAAGAGCATGGAC | CGAGTCATCGTATTCGAGCGG |
| VWDE | CTGAAACAGGAGGTGATTGTGT | GCTATGTGAAATCCCACTGAGTT |
| Twist1 | AAGATCATCCCCACGCTGC | CTCCATCCTCCAGACGGAGA |
| ADAMTS1 | CAGAGCACTATGACACAGCAA | AGCCATCCCAAGAGTATCACA |
| FGFR2 | GGAAAGTGTGGTCCCATCTGA | TCCAGGTGGTACGTGTGATTG |
| SFR5 | TGCTGCACTGCCACAAGTT | GTGCTCCATCTCACACTGGG |
| Wnt4 | CTCCACACTCGACTCCTTGC | CCGAAGAGATGGCGTACACG |
| DKK1 | CCTTGAACTCGGTTCTCAATTCC | CAATGGTCTGGTACTTATTCCCG |
| SOX6 | GGATGCAATGACCCAGGATTT | TGAATGGTACTGACAAGTGTTGG |
| LEF1 | TGCCAAATATGAATAACGACCCA | GAGAAAAGTGCTCGTCACTGT |
| TCF | CACGGGCAAACACTACGGT | TTGACCTTCGAGTGCTGATCC |
| FOXA1 | GCAATACTCGCCTTACGGCT | TACACACCTTGGTAGTACGCC |
| CCND3 | TACCCGCCATCCATGATCG | AGGCAGTCCACTTCAGTGC |
