# Supplementary material for: Knockdown of CD146 promotes endothelial-to-mesenchymal transition via Wnt/β-catenin pathway
Source: PLoS One. 2022 Aug 24;17(8):e0273542. doi: 10.1371/journal.pone.0273542 (PMC9401105; doi:10.1371/journal.pone.0273542)
Supplement: S1 Fig — Mice were subjected to Ang Ⅱ (1.4 mg/kg/day) infusion for 2 (A) and 4 (B) weeks. Blood pressure was measured under conscious state by the noninvasive tail-cuff method using the CODA blood pressure system on the day of surgery and at 7, 14, 21, 28 days after surgery. *P<0.05 vs. 0 day, **P<0.01 vs. 0 day. (DOCX) [file pone.0273542.s002.docx]

**S1 Fig.**

**A**

**B**
