# Supplementary figures and images for: Knockdown of CD146 promotes endothelial-to-mesenchymal transition via Wnt/β-catenin pathway
Source: PLoS One. 2022 Aug 24;17(8):e0273542. doi: 10.1371/journal.pone.0273542 (PMC9401105; doi:10.1371/journal.pone.0273542)

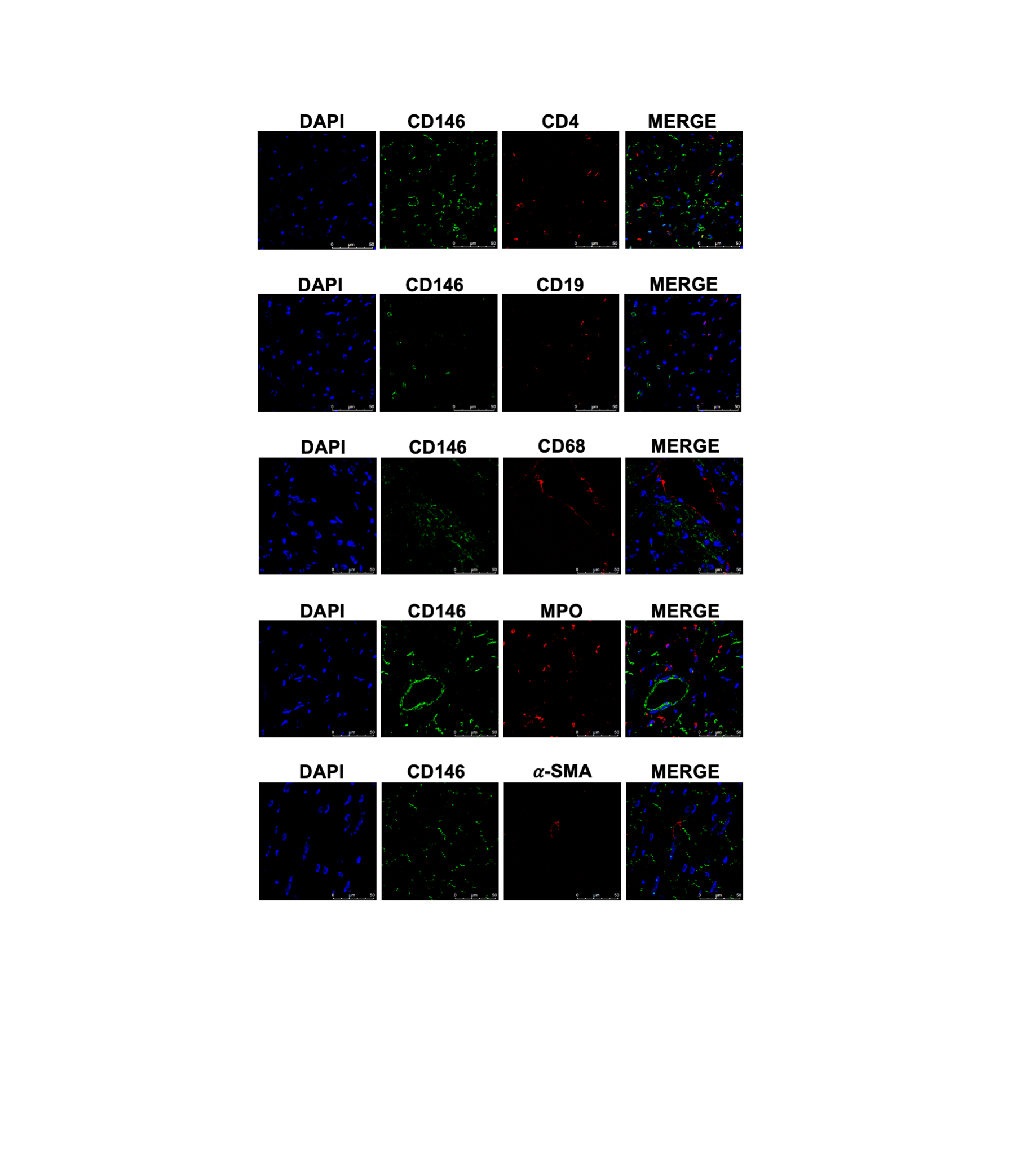

Supplement: S2 Fig — Representative double-immunofluorescence images of CD146 with α-SMA (a marker for SMCs), MPO (a marker for neutrophils), CD68 (a marker for monocytes/macrophages), CD19 (a marker for B lymphocytes), and CD4 (a marker for T-helper cells) in left ventricles from adult mice (scale bars indicate 50μm). Green represents CD146; red represents CD4, CD19, CD68, MPO and α-SMA, respectively; blue represents nuclei. (TIF) [file pone.0273542.s003.tif]
