# Supplementary material for: Knockdown of CD146 promotes endothelial-to-mesenchymal transition via Wnt/β-catenin pathway
Source: PLoS One. 2022 Aug 24;17(8):e0273542. doi: 10.1371/journal.pone.0273542 (PMC9401105; doi:10.1371/journal.pone.0273542)
Supplement: S1 Raw images — (PDF) [file pone.0273542.s005.pdf]

Fig 1B

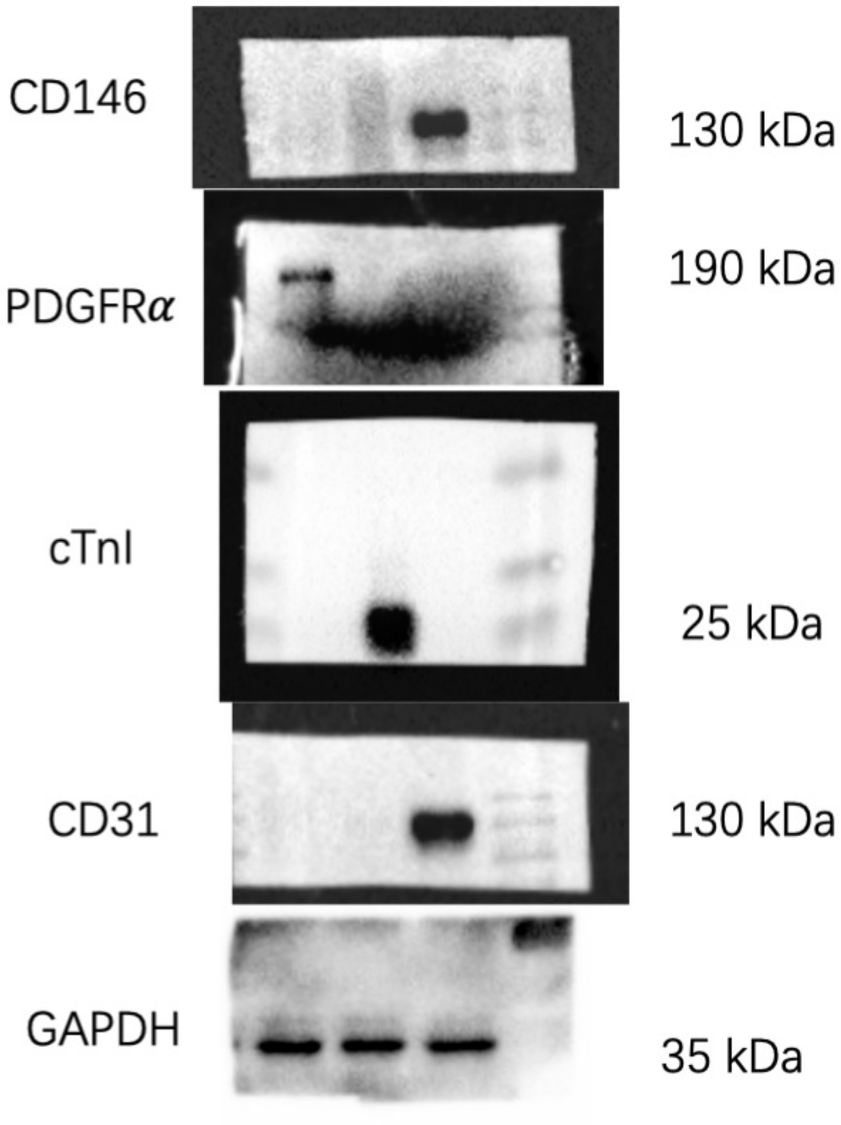
CD146
130 kDa
PDGFRα
190 kDa
cTnI
25 kDa
CD31
130 kDa
GAPDH
35 kDa

Fig 2

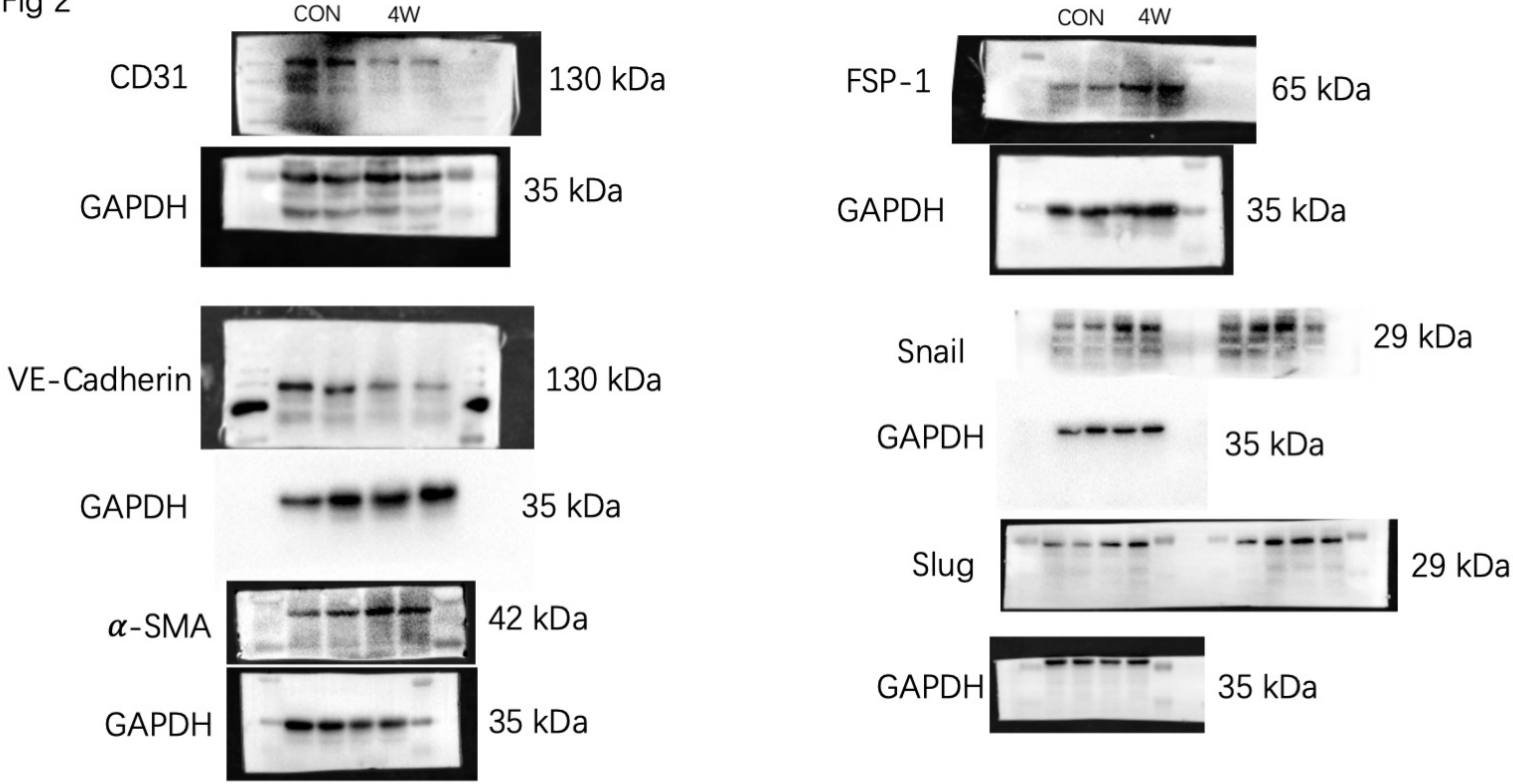
CON
4W
CD31
130 kDa
GAPDH
35 kDa
VE-Cadherin
130 kDa
GAPDH
35 kDa
α-SMA
42 kDa
GAPDH
35 kDa
CON
4W
FSP-1
65 kDa
GAPDH
35 kDa
Snail
29 kDa
GAPDH
35 kDa
Slug
29 kDa
GAPDH
35 kDa

Fig 2

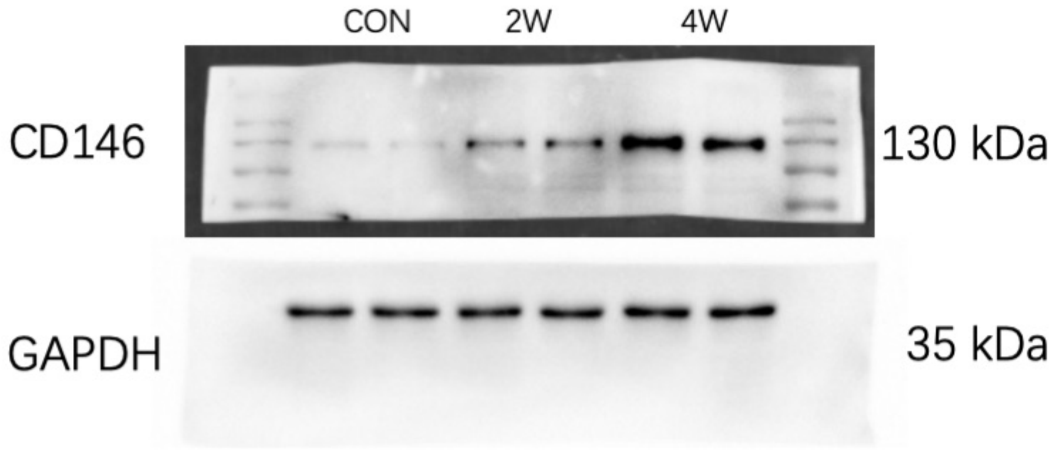

Fig 2C  For analysis

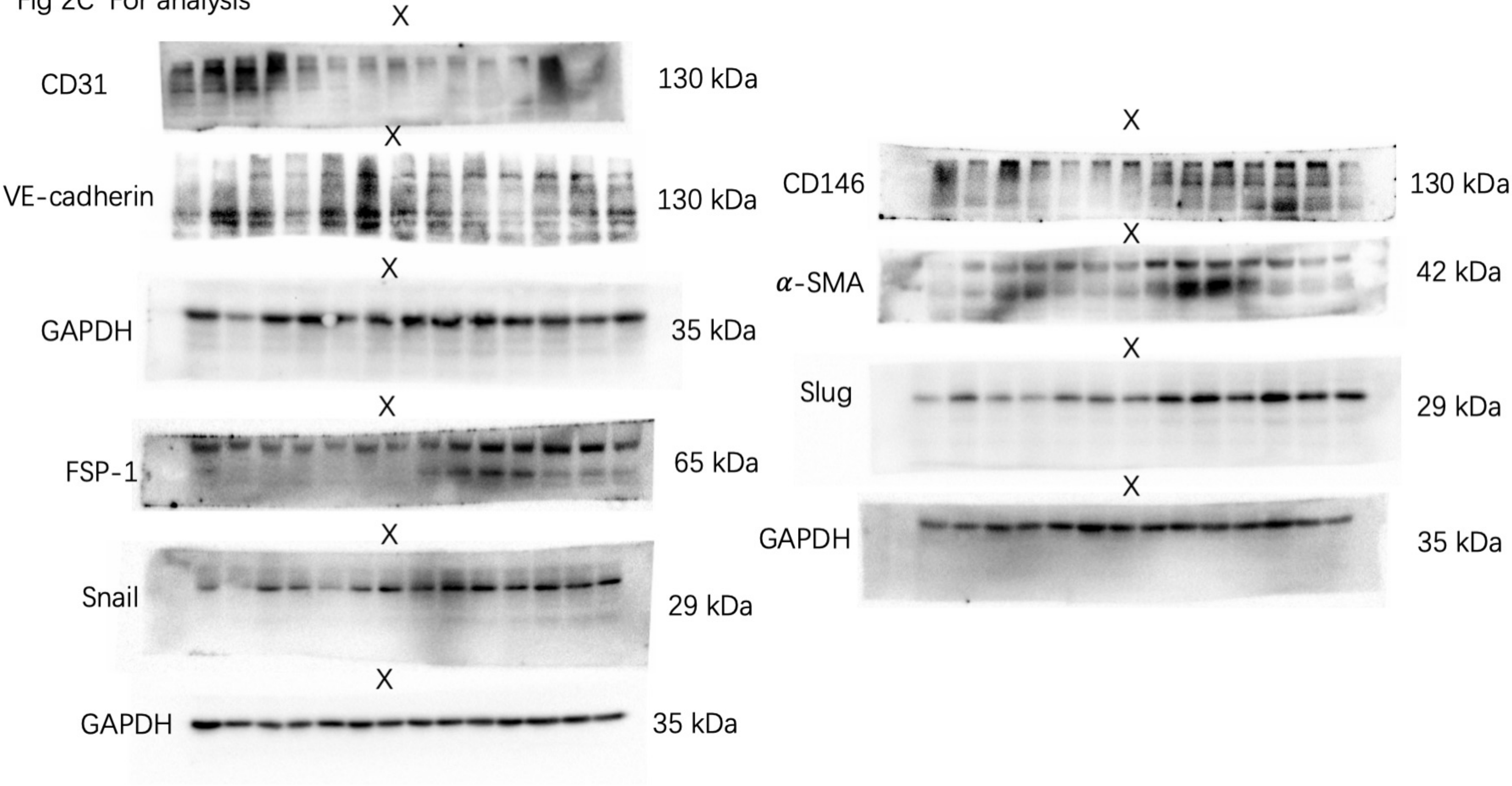

Fig 3D

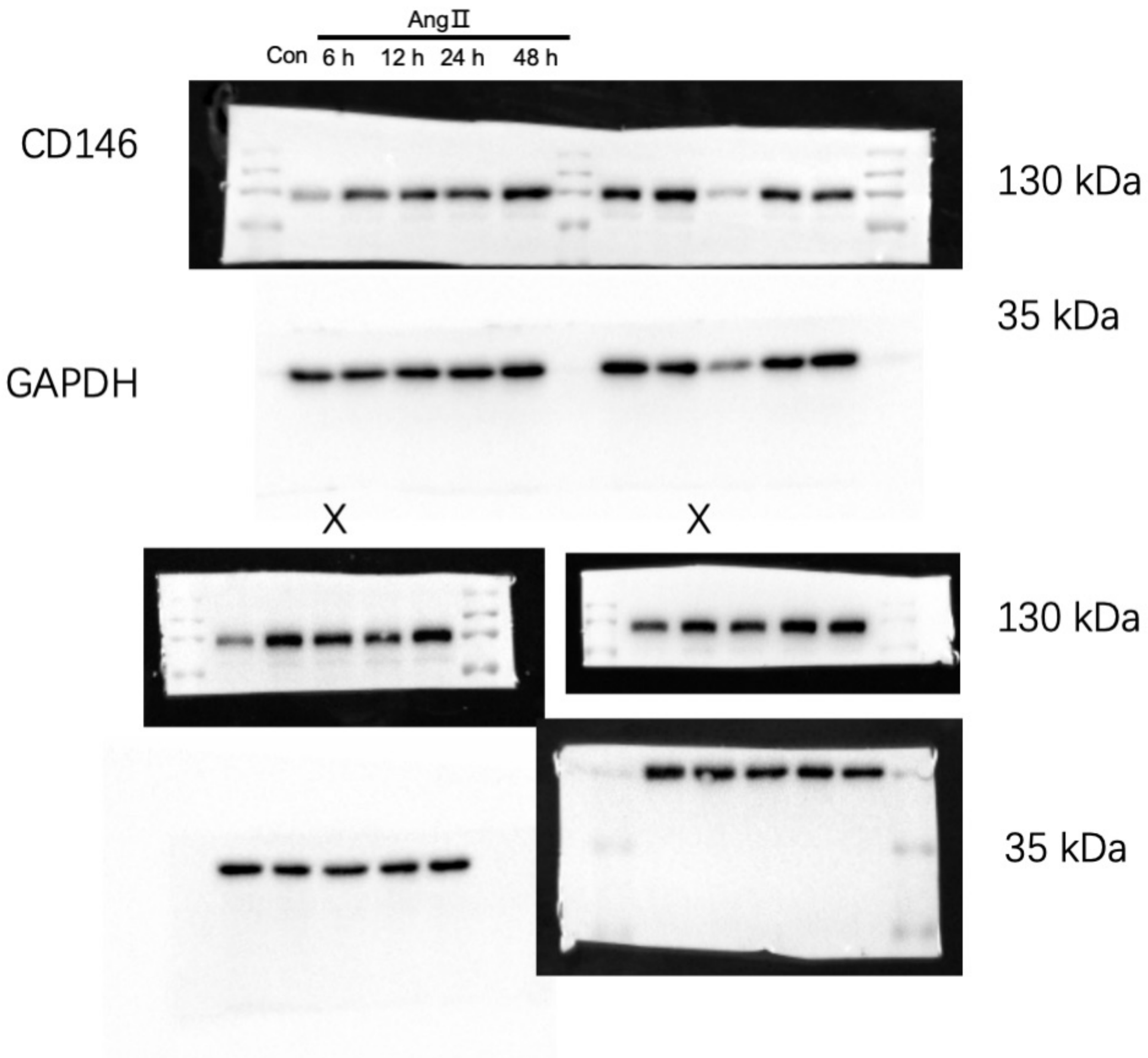
AngⅡ
Con
6 h
12 h
24 h
48 h
CD146
GAPDH
130 kDa
35 kDa
X
X
130 kDa
35 kDa

Fig 5

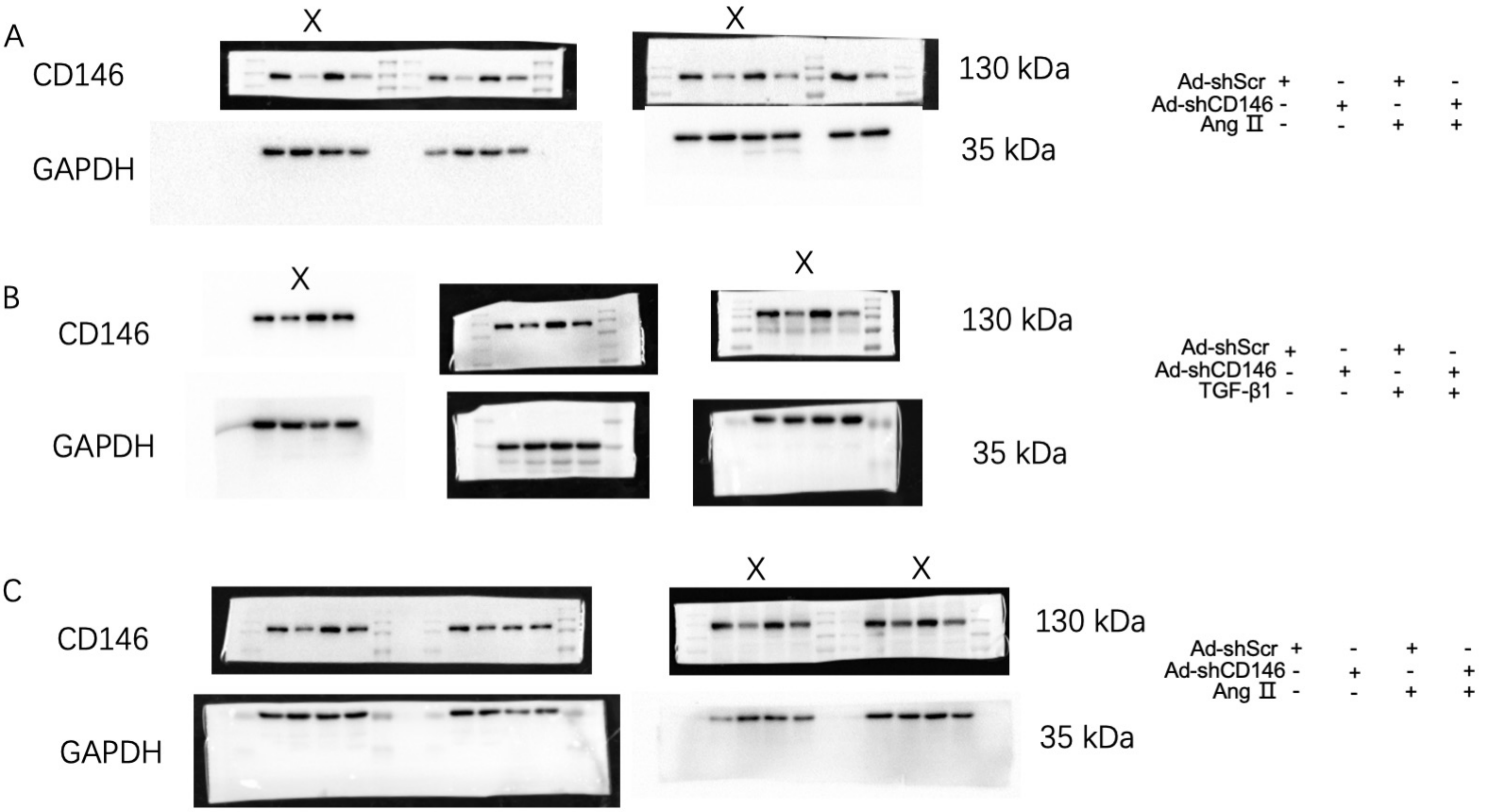
A
X
X
CD146
GAPDH
130 kDa
35 kDa
Ad-shScr + - + -
Ad-shCD146 - + - +
Ang Ⅱ - - + +
B
X
X
CD146
GAPDH
130 kDa
35 kDa
Ad-shScr + - + -
Ad-shCD146 - + - +
TGF-β1 - - + +
C
X
X
CD146
GAPDH
130 kDa
35 kDa
Ad-shScr + - + -
Ad-shCD146 - + - +
Ang Ⅱ - - + +

Fig 5

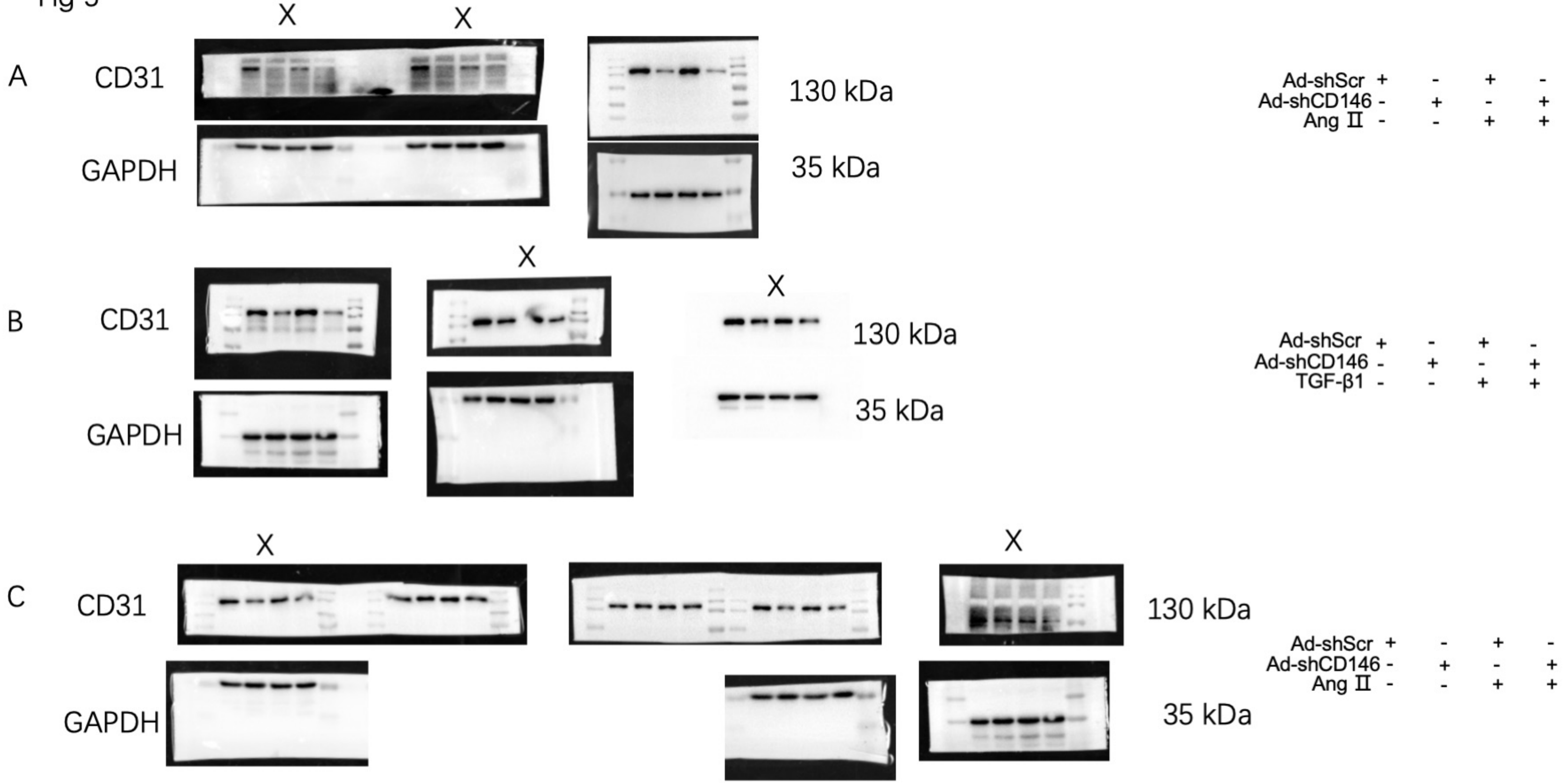
X
X
A
CD31
GAPDH
130 kDa
35 kDa
Ad-shScr + - + -
Ad-shCD146 - + - +
Ang Ⅱ - - + +
X
X
B
CD31
GAPDH
130 kDa
35 kDa
Ad-shScr + - + -
Ad-shCD146 - + - +
TGF-β1 - - + +
X
X
C
CD31
GAPDH
130 kDa
35 kDa
Ad-shScr + - + -
Ad-shCD146 - + - +
Ang Ⅱ - - + +

Fig 5

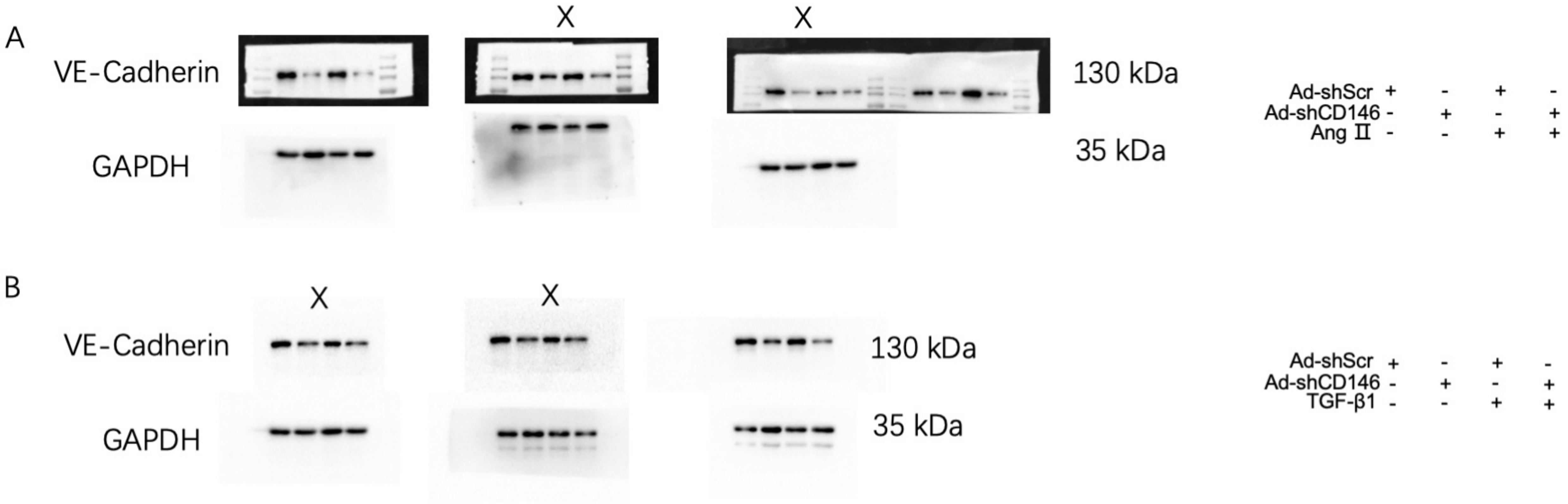
A
X
X
VE-Cadherin
GAPDH
130 kDa
35 kDa
Ad-shScr + - + -
Ad-shCD146 - + - +
Ang II - - + +
B
X
X
VE-Cadherin
GAPDH
130 kDa
35 kDa
Ad-shScr + - + -
Ad-shCD146 - + - +
TGF-β1 - - + +

Fig 5

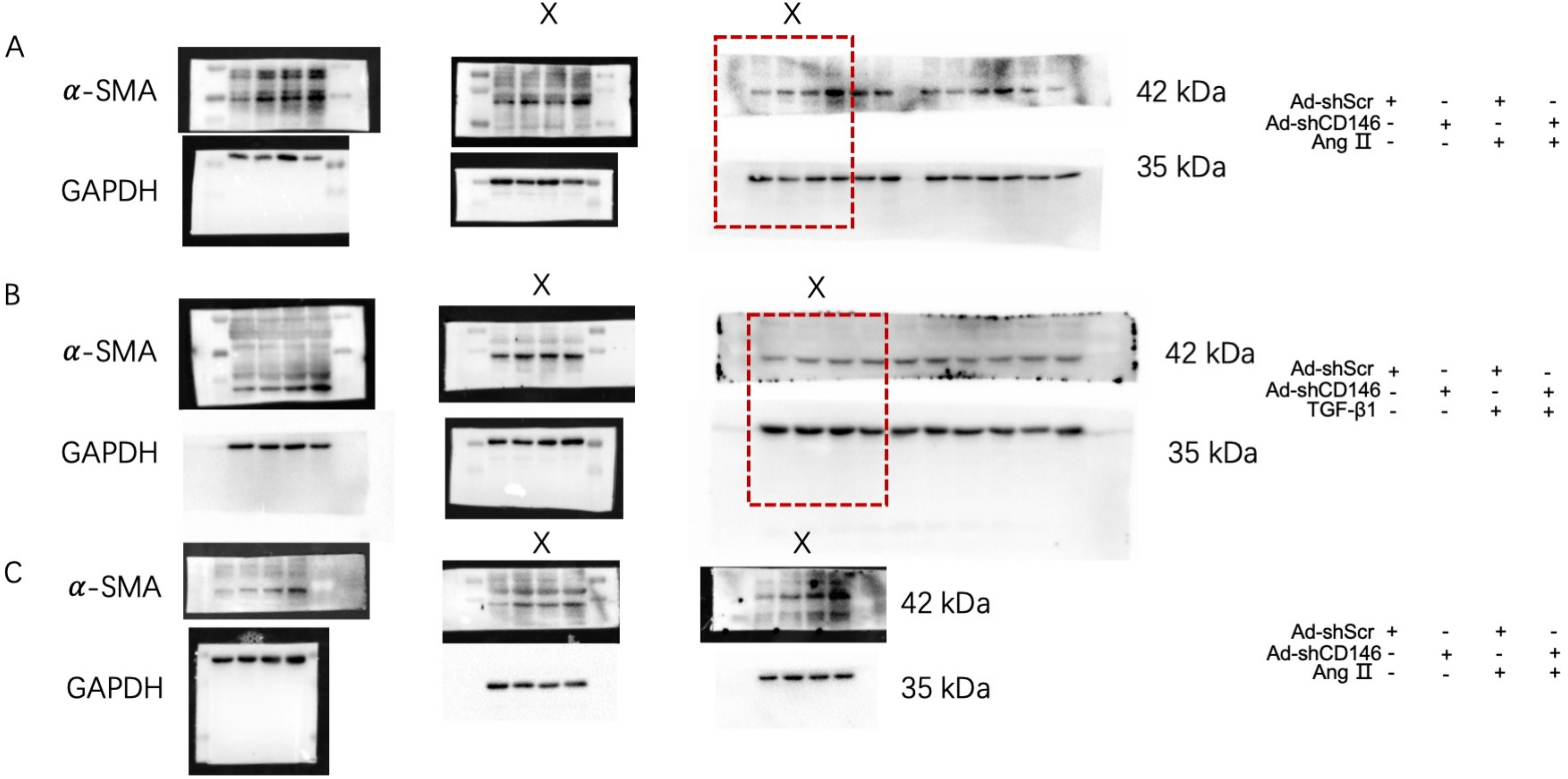

Fig 5

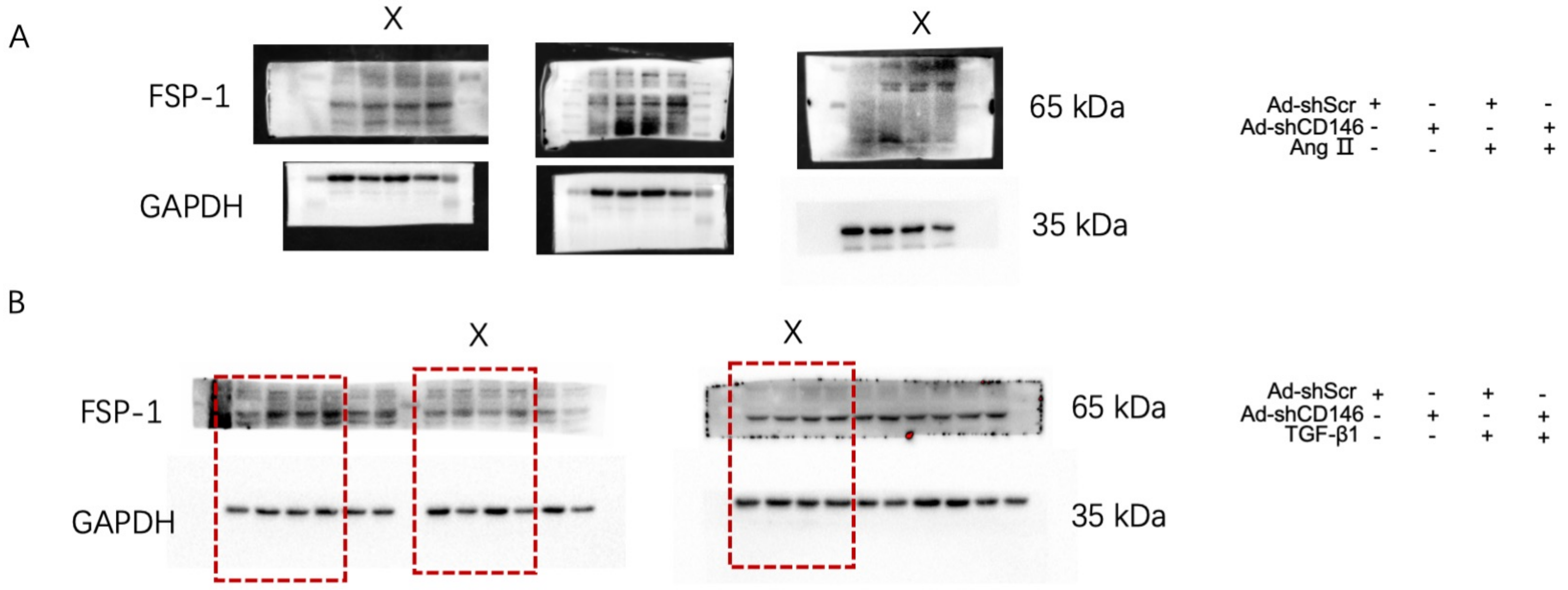
A
X
X
FSP-1
GAPDH
65 kDa
35 kDa
Ad-shScr + - + -
Ad-shCD146 - + - +
Ang II - - + +
B
X
X
FSP-1
GAPDH
65 kDa
35 kDa
Ad-shScr + - + -
Ad-shCD146 - + - +
TGF-β1 - - + +

Fig 5

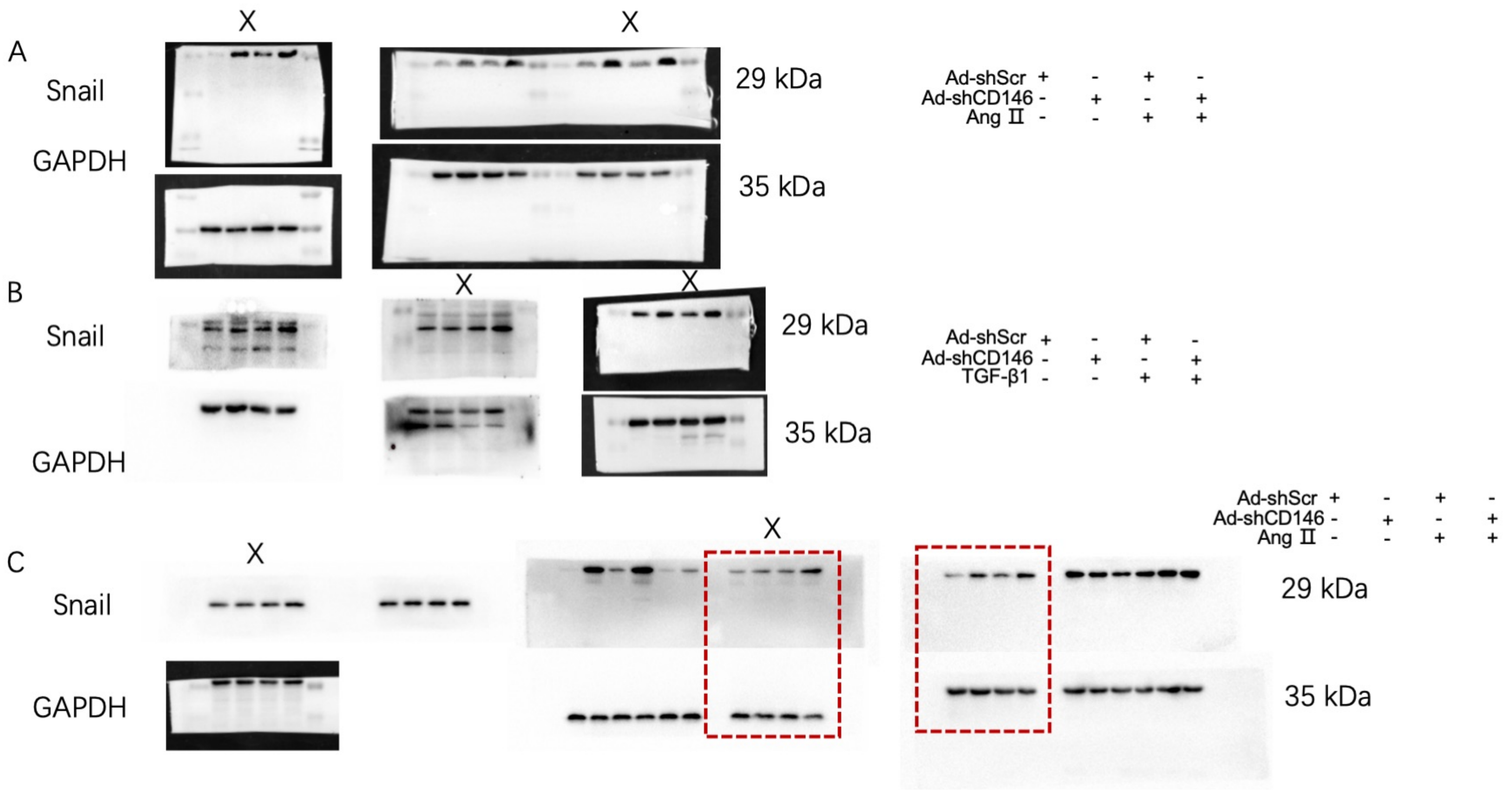

Fig 5

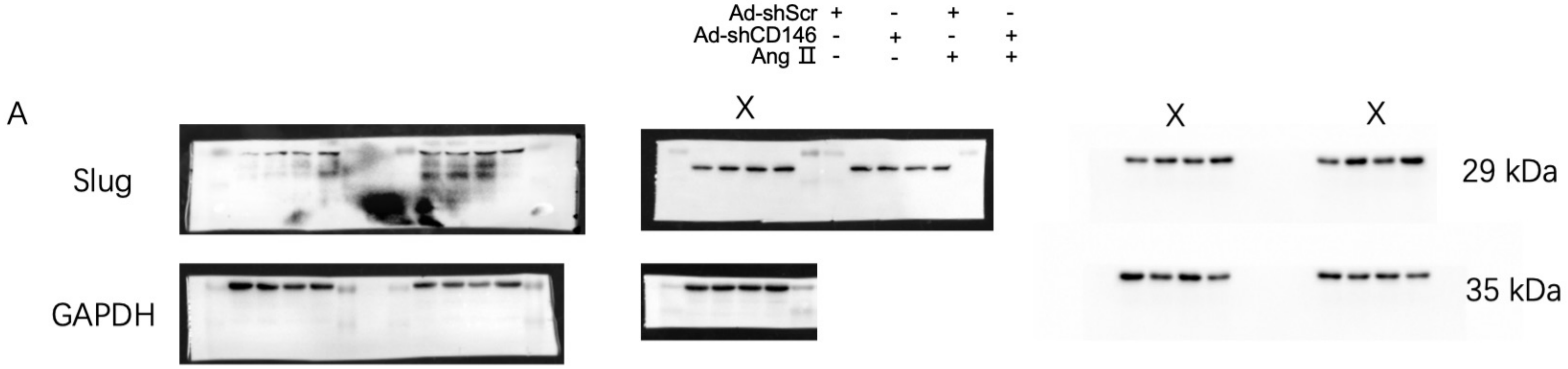

Fig 6

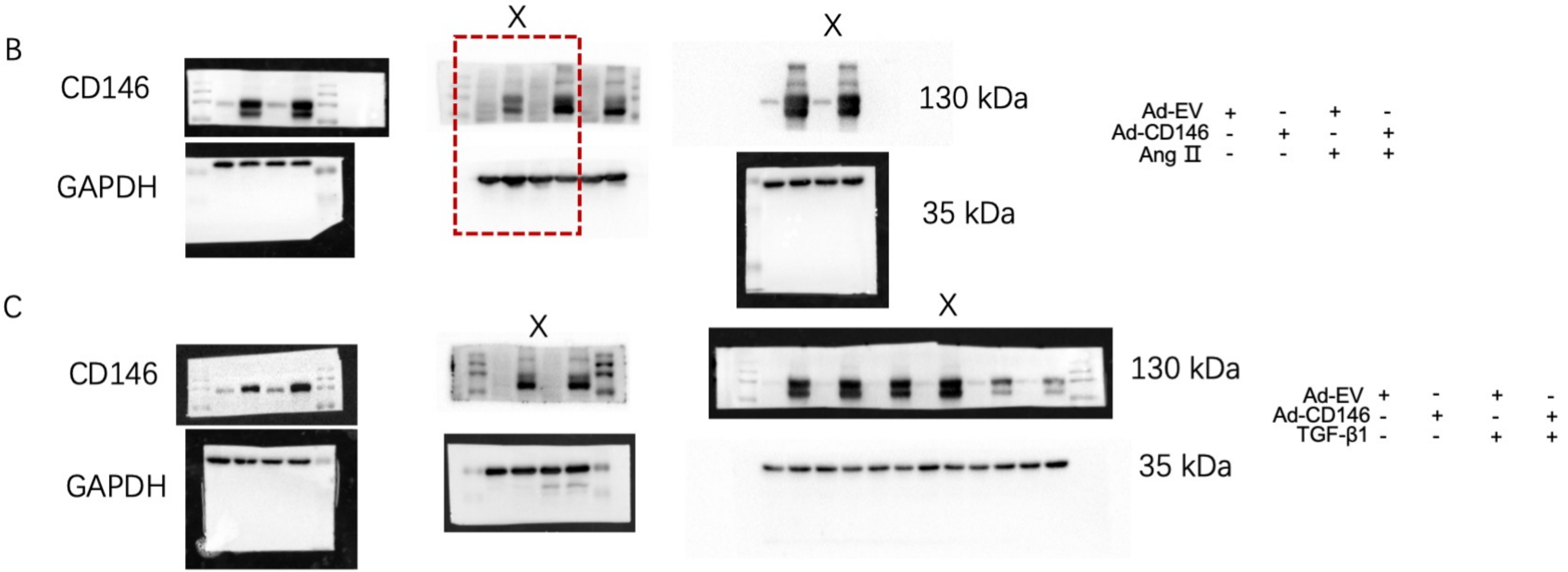
B
CD146
GAPDH
X
X
130 kDa
35 kDa
Ad-EV + - + -
Ad-CD146 - + - +
Ang Ⅱ - - + +
C
CD146
GAPDH
X
X
130 kDa
35 kDa
Ad-EV + - + -
Ad-CD146 - + - +
TGF-β1 - - + +

Fig 6

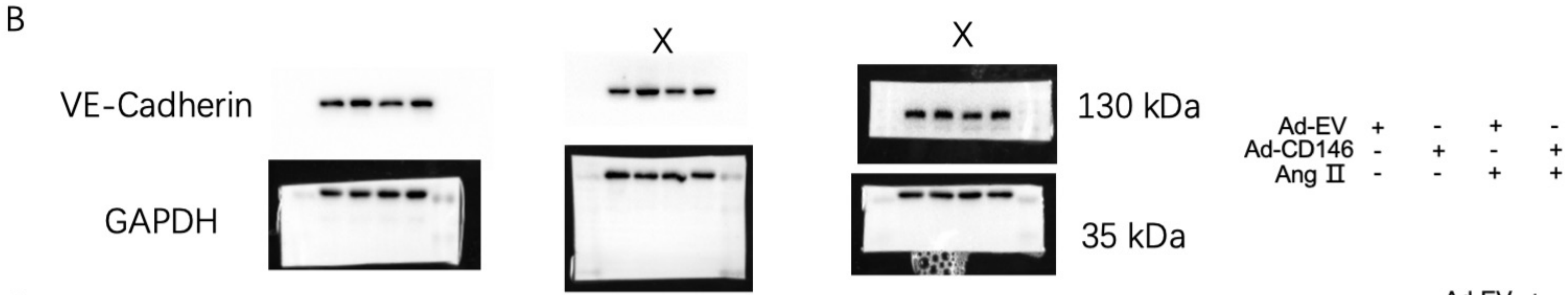
B
X
X
VE-Cadherin
GAPDH
130 kDa
35 kDa
Ad-EV + - + -
Ad-CD146 - + - +
Ang Ⅱ - - + +

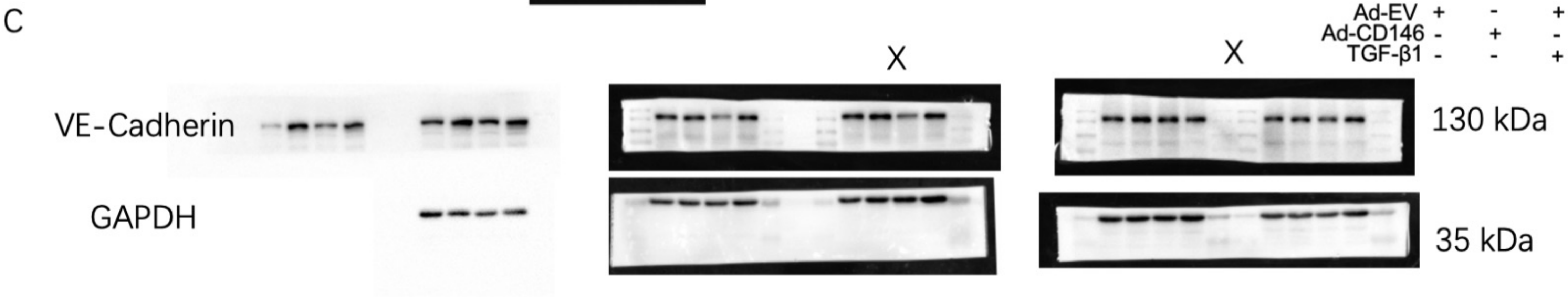
C
Ad-EV + - + -
Ad-CD146 - + - +
TGF-β1 - - + +
X
X
VE-Cadherin
GAPDH
130 kDa
35 kDa

Fig 6

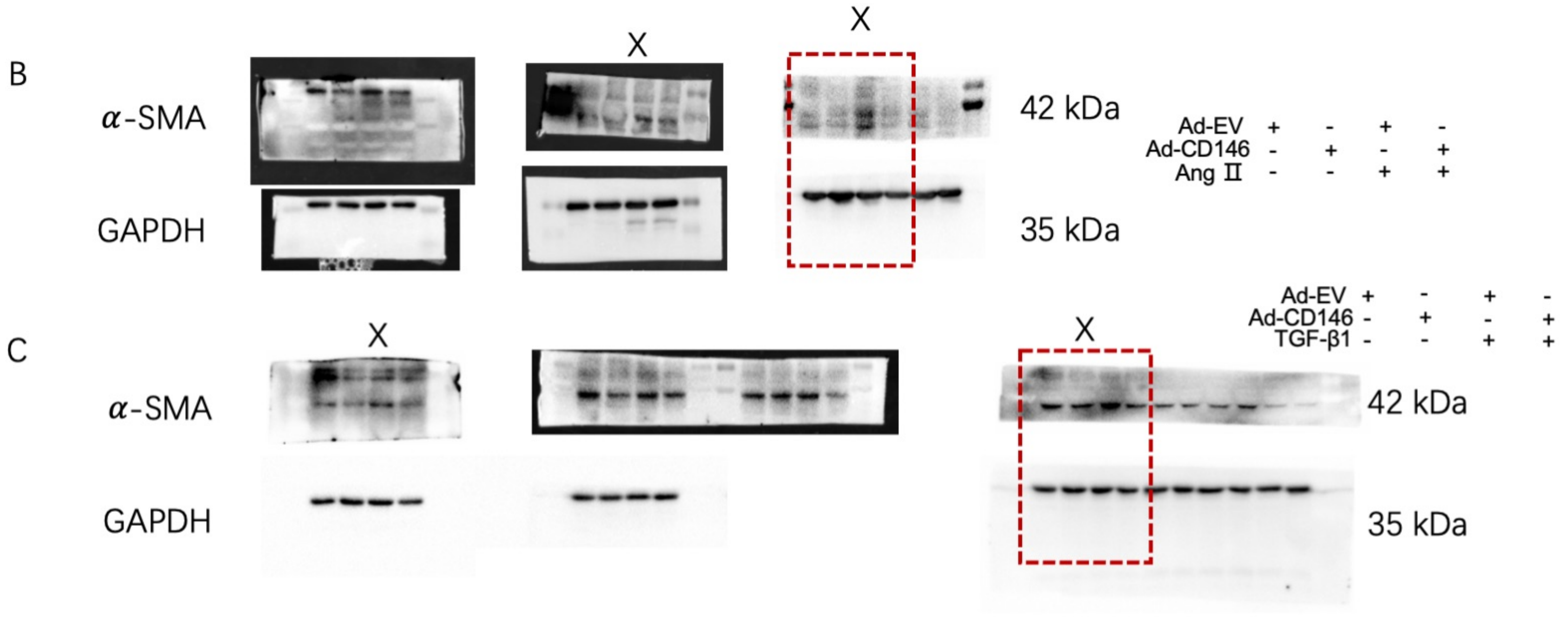
B
X
X
α-SMA
GAPDH
42 kDa
35 kDa
Ad-EV + - + -
Ad-CD146 - + - +
Ang Ⅱ - - + +
C
X
X
Ad-EV + - + -
Ad-CD146 - + - +
TGF-β1 - - + +
α-SMA
GAPDH
42 kDa
35 kDa

Fig 6

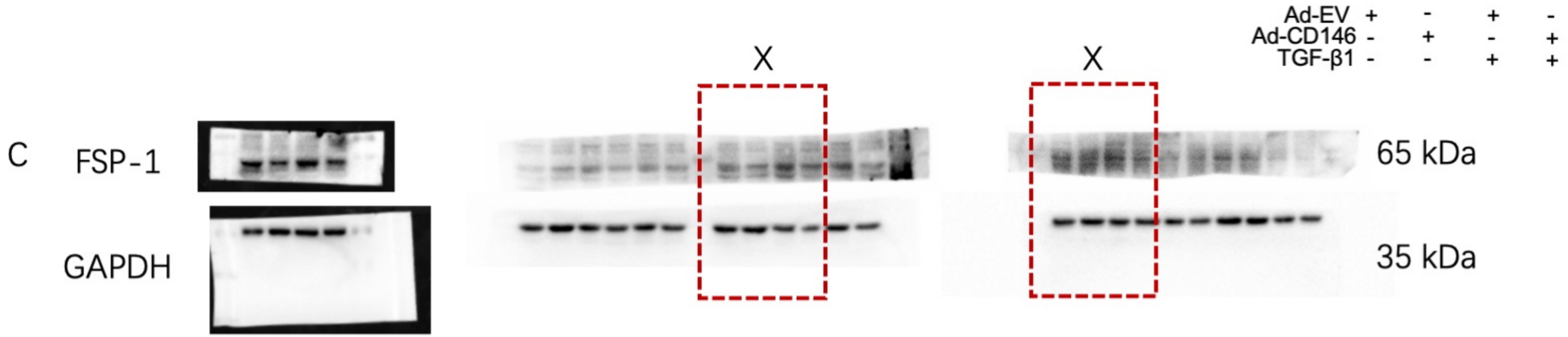

Fig 6

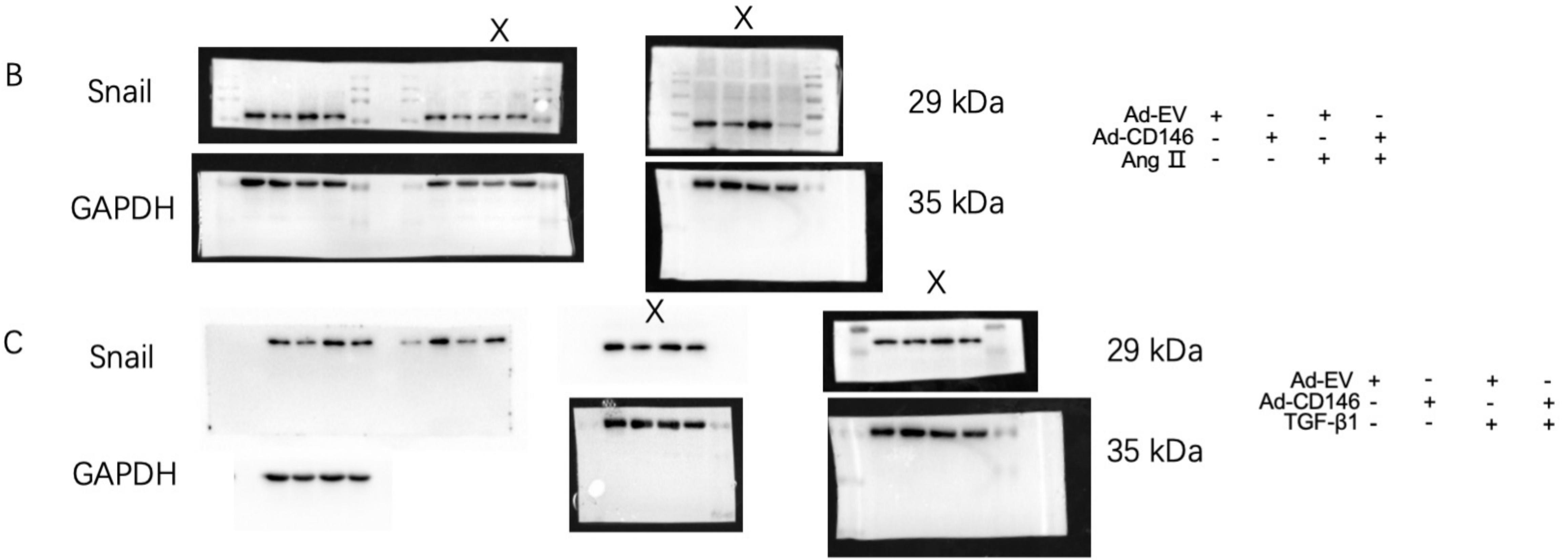
B
Snail
GAPDH
X
X
29 kDa
35 kDa
Ad-EV + - + -
Ad-CD146 - + - +
Ang Ⅱ - - + +
C
Snail
GAPDH
X
X
29 kDa
35 kDa
Ad-EV + - + -
Ad-CD146 - + - +
TGF-β1 - - + +

Fig 7

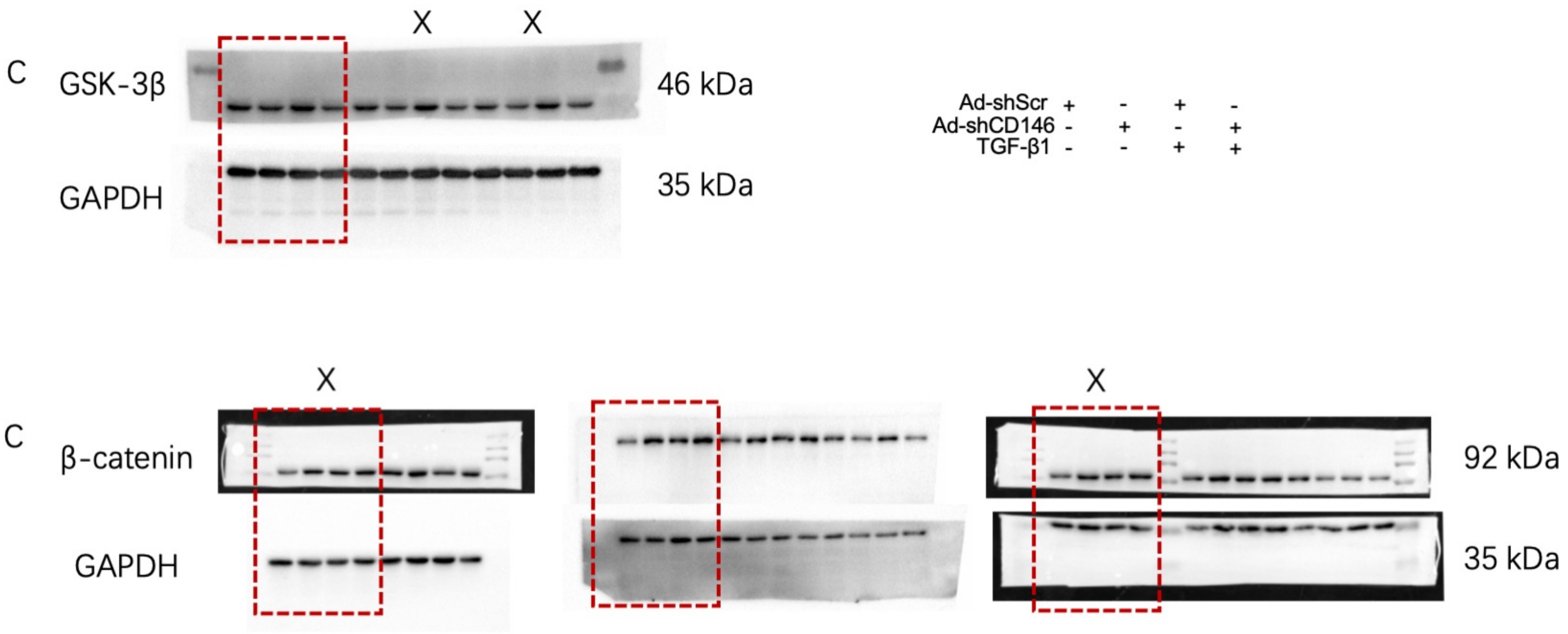
X
X
C
GSK-3β
46 kDa
GAPDH
35 kDa
Ad-shScr + - + -
Ad-shCD146 - + - +
TGF-β1 - - + +
X
X
C
β-catenin
92 kDa
GAPDH
35 kDa
